# Ethnic disparities in mortality from acute coronary syndromes: a systematic review and meta-analysis

**DOI:** 10.1136/openhrt-2026-004072

**Published:** 2026-04-30

**Authors:** Yasmin Mayet, Devan Wasan, Dario Sesia, Anoop S V Shah, Thomas Johnson, Amit Kaura

**Affiliations:** 1 University of Bristol, Bristol, UK; 2 Faculty of Medicine, Imperial College London, London, UK; 3 Imperial College Healthcare NHS Trust, London, UK; 4 London School of Hygiene and Tropical Medicine, London, UK; 5 NIHR Bristol Biomedical Research Centre, Bristol, UK; 6 Department of Cardiology, Imperial College London, London, UK

**Keywords:** Acute Coronary Syndrome, Myocardial Infarction, Systematic Reviews as Topic, Meta-Analysis

## Abstract

**Objective:**

To quantify ethnic disparities in mortality after acute coronary syndrome (ACS) by comparing outcomes in Black, Asian and hite population groups.

**Methods:**

We conducted a systematic review and meta-analysis of observational studies reporting mortality after ACS by ethnicity. Embase, Global Health, Ovid MEDLINE and Web of Science were searched through to March 2026 for English-language studies of adults with ST-elevation myocardial infarction (STEMI), non-STEMI or unstable angina. Two reviewers independently screened records, extracted data and assessed risk of bias using the Newcastle-Ottawa Scale. The primary outcome was all-cause mortality. Risk ratios (RRs), ORs and HRs were pooled as relative risk (RRs) with 95% CIs using random-effects models. Heterogeneity was quantified with I² and explored using prespecified subgroup analyses and meta-regression.

**Results:**

Forty cohort and registry studies from the USA, UK and Canada including 14 million patients met the inclusion criteria. Overall mortality was similar in black versus white patients (RR 0.99, 95% CI 0.94 to 1.03) and Asian versus white patients (RR 1.06, 95% CI 0.95 to 1.17); however, restriction to US-based studies demonstrated higher mortality in Asian patients (RR 1.14, 95% CI 1.03 to 1.27). Subgroup analyses showed higher mortality in black patients following STEMI (RR 1.09, 95% CI 1.02 to 1.17). Meta-regression showed age-dependent effect modification in black versus white comparisons, with differences attenuating in older populations. The pooled risk of major bleeding was similar between groups.

**Conclusions:**

Black patients had higher mortality than white patients following STEMI, and Asian patients demonstrated higher mortality in US-based studies. Overall post-ACS mortality was otherwise similar across ethnic groups. These findings suggest disparities persist in specific contexts and may be more pronounced in younger populations, but should be interpreted with caution due to substantial heterogeneity.

**PROSPERO registration number:**

https://www.crd.york.ac.uk/PROSPERO/view/CRD420250465260

**WHAT IS ALREADY KNOWN ON THIS TOPIC**

- Ethnic disparities in the incidence, management and outcomes of coronary heart disease are well recognised, but evidence on mortality after acute coronary syndrome (ACS) by ethnicity is inconsistent and based largely on non-contemporary, moderate-sized cohorts with short follow-up and incomplete adjustment.

**WHAT THIS STUDY ADDS**

- This contemporary systematic review and meta-analysis synthesises data from 40 studies, representing 14.2 million patients across the UK, USA and Canada. Black patients experienced higher mortality than white patients following ST-elevation myocardial infarction (STEMI), while overall post-ACS mortality was similar between black and white patients. Although overall mortality was similar between asian and white patients across all included studies, asian patients demonstrated higher mortality than white patients in studies conducted in the USA. Age was a significant effect modifier, with differences in mortality between black and white patients attenuating in older populations.

**HOW THIS STUDY MIGHT AFFECT RESEARCH, PRACTICE OR POLICY**

- By identifying higher post-STEMI mortality in black patients and demonstrating that elevated mortality in asian patients may be context-dependent, this study highlights the need for research into the structural and clinical factors underlying these disparities. These findings may inform more equitable access to timely reperfusion, invasive management, rehabilitation and secondary prevention for black and asian populations, and underscore the importance of using granular ethnicity data in future registry studies and clinical audits.

## Introduction

Ischaemic heart disease is a leading cause of mortality worldwide, with acute coronary syndromes (ACS) representing one of its most severe manifestations. ACS includes ST-elevation myocardial infarction (STEMI), non-STEMI (NSTEMI) and unstable angina, and is characterised by a sudden reduction in myocardial blood flow. It is estimated to account for approximately 17 million deaths each year.^1^

There are well-documented disparities in the incidence, management and outcomes of ACS by ethnicity. Although ACS incidence has declined in many high-income settings, improvements appear greater in white populations than in some ethnic groups, including black patients.^2 3^ Contributing factors may include differences in cardiovascular risk profiles, socio-economic and cultural influences, institutional and structural factors, provider bias and health-seeking behaviours.^4^

Observational studies suggest ethnic differences in the investigation and treatment of coronary artery disease. Black patients have lower rates of revascularisation and are less likely to be admitted for ACS or to receive invasive management than white patients.^5 6^ However, studies examining ethnic differences in mortality after ACS report mixed findings, with some describing higher mortality in black patients^7^,^10^ and others reporting little or no difference.^2 7^ Many studies are limited by small sample sizes, short follow-up or incomplete adjustment.

Only one prior meta-analysis has compared mortality between black and white patients after ACS, but it predates recent changes in clinical practice, includes limited data on asian populations, and does not incorporate large contemporary registry studies published since 2022, including a UK-based cohort of over 400 000 patients.^8^

This study systematically reviews evidence on ethnic differences in outcomes after ACS, evaluating mortality among white European, black and asian patients, and examining secondary outcomes including major adverse cardiac events, cardiac mortality, all-cause mortality, reinfarction and major bleeding.

## Methods

### Study design and reporting framework

We conducted a systematic review and meta-analysis of observational studies comparing outcomes after ACS between african Caribbean, asian and white European patients. The review was designed and reported in accordance with the MOOSE (Meta-analysis Of Observational Studies in Epidemiology) guidelines and the PRISMA (Preferred Reporting Items for Systematic Reviews and Meta-Analyses) statement.^9 10^ A prespecified protocol was registered on PROSPERO (CRD420250465260), outlining eligibility criteria, outcomes and analytical approach.

### Information sources and search strategy

We searched Embase, Global Health, Ovid MEDLINE and Web of Science from database inception to March 2026. Search strategies combined controlled vocabulary and free-text terms related to ethnicity or race, ACS and clinical outcomes. Ethnicity-related terminology was standardised across databases using both controlled vocabulary terms (such as MeSH headings for ethnic groups and continental ancestry groups) and a broad range of free-text synonyms encompassing self-identified ethnic labels used in the UK, USA and Canadian contexts, including black, African American, Afro-Caribbean, Asian, South Asian, East Asian, and white or Caucasian, among others. The search was structured as four concept blocks: (1) coronary revascularisation and invasive or conservative management strategies; (2) ACS and related diagnoses; (3) clinical outcomes including mortality, major adverse cardiovascular events, reinfarction and major bleeding; and (4) ethnicity and racial identity terms, combining two complementary ethnicity blocks using OR. The four blocks were combined using AND. Reference lists of all included studies were screened for additional eligible studies. References specifically highlighted during the peer review process were also assessed for eligibility. Full database-specific search strategies are provided in the online supplemental Methods.

### Study selection

**Figure 1:**
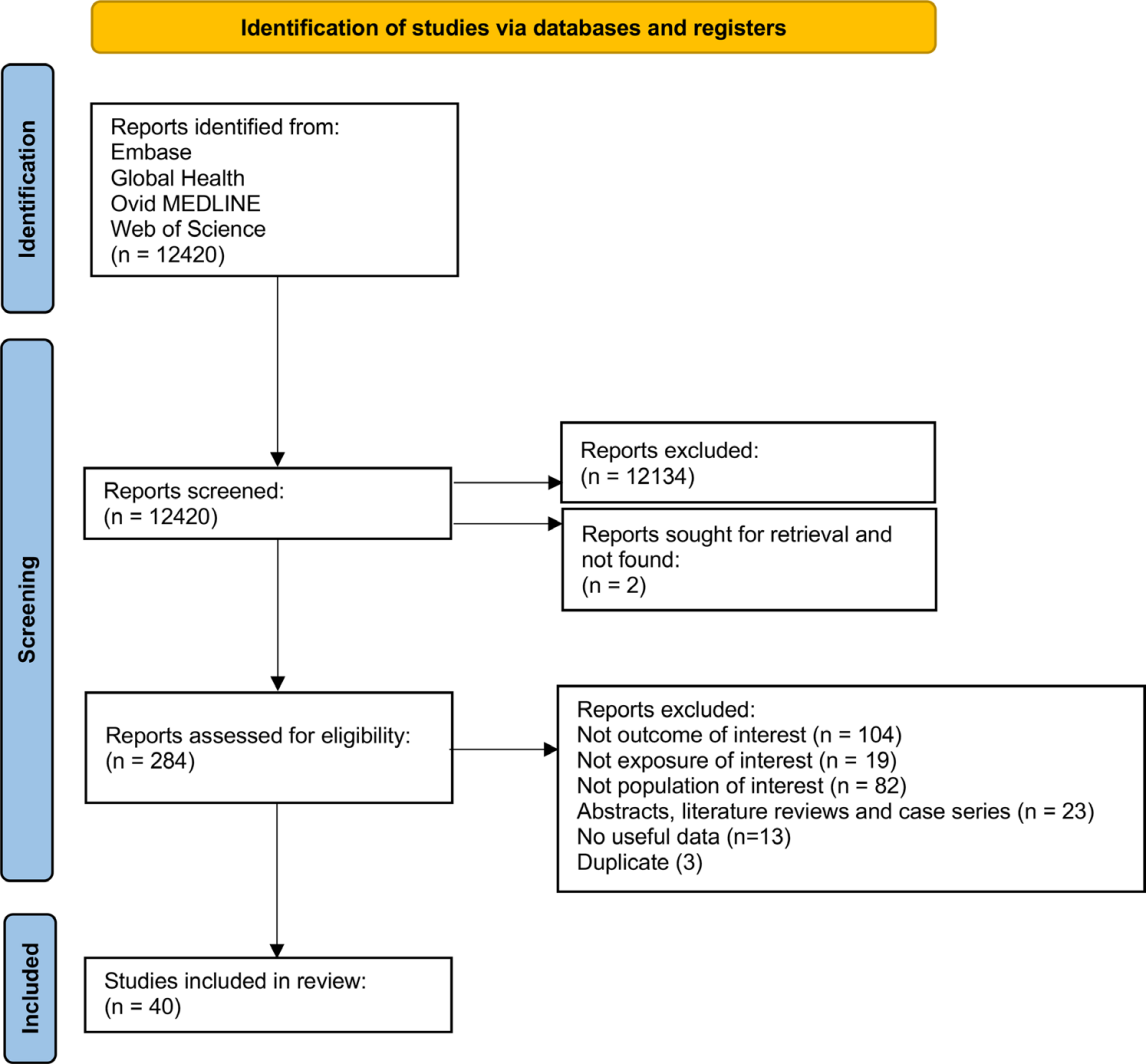
PRISMA 2020 flow diagram of study selection. PRISMA, Preferred Reporting Items for Systematic Reviews and Meta-Analyses.

All records were imported into Microsoft Excel and duplicates removed.^11^ Two reviewers (YM, DW) independently screened titles and abstracts and assessed full texts against inclusion and exclusion criteria (online supplemental table 2). Disagreements were resolved through consultation with a third reviewer (AK). The study selection process is summarised in a PRISMA flow diagram (figure 1).^12^

Studies were excluded if they reported outcomes exclusively in patients with ACS complicated by cardiogenic shock, cardiac arrest, stroke, intracranial haemorrhage and left ventricular aneurysm as these represent highly selected subpopulations in whom post-ACS mortality may be driven primarily by the acute complication rather than by ethnic differences in underlying care or prognosis. Studies were also excluded if the entire study population had undergone coronary revascularisation (percutaneous coronary intervention (PCI) or coronary artery bypass grafting), as these intervention-specific cohorts may not be representative of outcomes across the broader ACS population.

### Data extraction and management

Data were extracted independently by two reviewers (YM, DW) using a standardised, piloted form. Extracted variables are summarised in online supplemental table 1. Ethnicity definitions varied considerably across included studies, and most did not specify whether classification was self-reported or administratively assigned. This variability was noted and considered in the interpretation of pooled estimates.

### Outcomes and effect measures

The primary outcome was all-cause mortality after ACS, assessed at the longest reported follow-up. Secondary outcomes included major adverse cardiac events (as defined by individual studies), cardiac mortality, mortality at specific time points (eg, in-hospital, 30-day, 1 year), reinfarction and major bleeding. Where multiple adjusted models were reported, estimates from the most fully adjusted model were extracted. For studies reporting multiple time points, estimates from the longest follow-up were used for primary analyses. When necessary, data were extracted from tables, figures and supplementary material.

### Subgroup and sensitivity analysis

Subgroup and secondary analyses were conducted only when three or more studies with comparable exposure and reference ethnicities were available. Prespecified subgroup analyses examined outcomes by ACS subtype (STEMI vs NSTEMI), country of study, study decade and sex. Subgroup comparisons were performed for black versus white and asian versus white patients where sufficient data existed.

Random-effects meta-regression examined whether mean age and year of publication modified associations between ethnicity and mortality, using study-level covariates and log risk ratios as the outcome.

### Risk of bias assessment in individual studies

Risk of bias was assessed using the Newcastle-Ottawa Scale (NOS) for cohort studies.^13^ Two reviewers (YM, DW) independently rated each study, resolving discrepancies by discussion. NOS scores informed interpretation and sensitivity analyses.

### Data synthesis and statistical analysis

Given anticipated heterogeneity in populations, ethnicity definitions, clinical settings and adjustment sets, random-effects meta-analysis was used. Analyses were conducted in R V.4.4.1 using standard methods for observational meta-analysis.^14^ Log-transformed risk ratios and SEs were pooled, with between-study variance (τ²) estimated.

Risk ratios, ORs and HRs were extracted. For meta-analysis, ORs and HRs were treated as approximations to risk ratios when outcomes were uncommon and effects small. This approach is considered acceptable when outcome events are not common and effect sizes are modest, as the OR and HR approximate the risk ratios under these conditions. Where mortality rates were high or follow-up prolonged, this approximation introduces greater uncertainty, and findings from such studies are interpreted accordingly.

For one study reporting an effect estimate and p value only, SEs and 95% CIs were derived using standard formulae (online supplemental Appendix 2).

Two primary meta-analyses were conducted: black versus white patients and asian versus white patients. Where studies reported multiple estimates for the same comparison, within-study estimates were first combined using fixed-effect methods.

**Figure 2:**
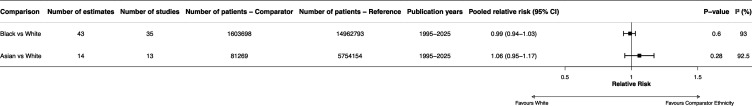
Pooled RRs for all-cause mortality after acute coronary syndrome comparing black versus white and Asian versus white populations. Estimates are shown as RRs with 95% CIs using random-effects models. I² quantifies heterogeneity; values >1 indicate higher mortality in the comparator group. RRs, relative risks.

Heterogeneity was quantified using Cochran’s Q, I² and τ². Forest plots summarised pooled estimates (figures2^4^). Publication bias and small-study effects were assessed using funnel plots and asymmetry tests where appropriate.

**Figure 3:**
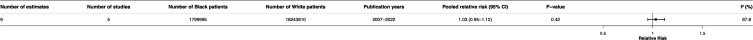
Pooled RR for major bleeding following acute coronary syndrome in black versus white patients. Estimates are presented as RRs with 95% CIs using random-effects models; I² indicates heterogeneity. RRs, relative risks.

**Figure 4:**
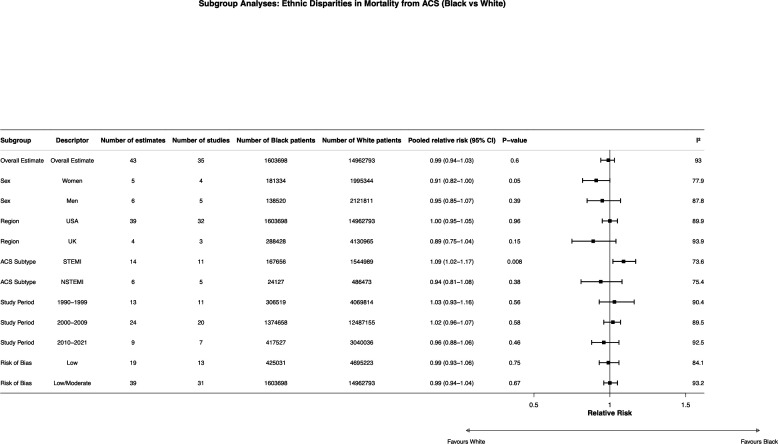
Subgroup analyses of pooled RRs for all-cause mortality following ACS in black versus white patients. Estimates are presented as RRs with 95% CIs using random-effects models; I² indicates heterogeneity. ACS, acute coronary syndrome; NSTEMI, non-ST-elevation myocardial infarction; RRs, relative risks; STEMI, ST-elevation myocardial infarction.

### Patient and public involvement

Patients and the public were not involved in the design, conduct, reporting or dissemination of this research. This study used previously published data, with no direct patient involvement.

## Results

### Study selection

Searches of Embase, Global Health, Ovid MEDLINE and Web of Science identified 12 420 records, all of which were screened at title and abstract level. Of these, 12 134 were excluded and two full texts could not be retrieved. The remaining 284 articles underwent full-text review; 244 were excluded because the outcome was not of interest (n=104), the population was not of interest (n=82), the exposure was not of interest (n=19), the article was an abstract, literature review or case series (n=23), no useful data could be extracted (n=13) or the article was a duplicate report (n=3). In total, 40 studies met the inclusion criteria and were included in the systematic review and meta-analysis. The study selection process is shown in the PRISMA 2020 flow diagram (figure 1).

### Study characteristics

Most studies were retrospective cohort or registry-based analyses, predominantly from the USA, with additional studies from the UK and Canada. Studies reporting mortality outcomes for black versus white patients included 35 studies encompassing 1 296 476 black patients and 12 788 010 white patients. Studies reporting mortality outcomes for asian versus white patients included 13 studies encompassing 110 991 asian patients. Sample sizes ranged from 316 to 4 045 267 participants, with mean ages ranging from 44.3 to 85.8 years. Study characteristics are summarised in online supplemental table 1, and variables adjusted for in multivariable models are presented in online supplemental table 3.

### Quantitative synthesis (meta-analysis)

The primary outcome was mortality, with substantial between-study heterogeneity across all analyses. Given this heterogeneity, pooled estimates should be interpreted as the average of a distribution of true effects rather than a single underlying effect size, and are presented with appropriate caution throughout.

For black versus white patients, 43 estimates from 35 studies yielded a pooled relative risk (RR) of 0.99 (95% CI 0.94 to 1.03; p=0.6; I²=93%; Figure 2), indicating no overall difference in mortality. For asian versus white patients, 14 estimates from 13 studies yielded a pooled RR of 1.06 (95% CI 0.95 to 1.17; p=0.28; I²=92.5%; Figure 2), also indicating no statistically significant overall difference, though with substantial heterogeneity across studies.

The key secondary outcome was major bleeding. Five studies reported bleeding outcomes for black versus white patients, with no significant difference observed (pooled RR 1.03, 95% CI 0.95 to 1.12; p=0.42; Figure 3).]

### Subgroup, sensitivity and additional analyses

To explore sources of heterogeneity, we conducted prespecified meta-regression analyses by year of publication and mean age for both black versus white and asian versus white comparisons. Age was a significant effect modifier for black versus white comparisons: for every 5-year increase in mean age, the RR for mortality decreased by approximately 4.9% (regression coefficient = −0.0088 per year; 95% CI −0.015 to −0.0029; p=0.0033; R² = 19.53%), indicating smaller ethnic differences in older populations. However, age was not a significant effect modifier for asian versus white comparisons, with no significant evidence of an association (regression coefficient=0.0085 per year; 95% CI −0.015 to 0.032; p=0.48; R² = 0.00%) (online supplemental figures 7 and 8).

Meta-regression by year of publication showed no evidence of effect modification in black versus white or asian versus white comparisons (black vs white: β=0.0005, p=0.85; asian vs white: β=0.0039, p=0.65, R² = 0%) (online supplemental figures 9 and 10).

### Sensitivity analyses by risk of bias

Of the 40 included studies, 15 were rated as low risk of bias, 21 as moderate risk and 5 as high risk using the NOS (online supplemental file 1). Restricting analyses to low and moderate risk studies, and to low-risk studies alone, produced similar estimates to the overall analysis for black versus white patients (low-risk only: RR 0.99, 95% CI 0.93 to 1.06; p=0.75; low-moderate risk: RR 0.99, 95% CI 0.94 to 1.04; p=0.67), indicating robustness of findings to risk of bias (figure 4). Similarly, for asian versus white patients, sensitivity analyses restricted to low-risk and moderate-risk studies produced consistent results (low-risk only: RR 1.02, 95% CI 0.83 to 1.24; p=0.88; low-moderate risk: RR 1.05, 95% CI 0.93 to 1.17; p=0.43).

*Subgroup analyses*

Subgroup analyses by sex for black versus white patients showed no statistically significant mortality difference for women (RR 0.91, 95% CI 0.82 to 1.00; p=0.05; five estimates, four studies) or men (RR 0.95, 95% CI 0.85 to 1.07; p=0.39; six estimates, five studies), though the result for women approached conventional thresholds for significance and should be interpreted cautiously given the small number of contributing studies (figure 4).

**Figure 5:**
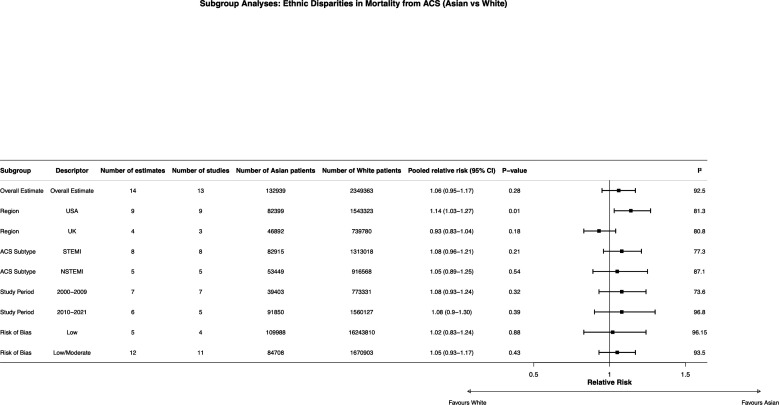
Subgroup analyses of pooled RRs for all-cause mortality following ACS in Asian versus white patients. Estimates are presented as RRs with 95% CIs using random-effects models; I² indicates heterogeneity. ACS, acute coronary syndrome; NSTEMI, non-ST-elevation myocardial infarction; RRs, relative risks; STEMI, ST-elevation myocardial infarction.

Restricting analyses to US-based studies produced results consistent with the overall meta-analysis for black versus white patients (RR 1.00, 95% CI 0.95 to 1.05; p=0.96; 39 estimates, 32 studies; Figure 4). For asian versus white patients, however, restriction to US-based studies demonstrated significantly higher mortality in asian patients (RR 1.14, 95% CI 1.03 to 1.27; p=0.01; nine estimates, nine studies; figure 5), while UK-based studies showed no significant difference (RR 0.93, 95% CI 0.83 to 1.04; p=0.18; four estimates, three studies).

Subgroup analyses by ACS subtype for black versus white patients showed significantly higher mortality in black patients following STEMI (RR 1.09, 95% CI 1.02 to 1.17; p=0.008; 14 estimates, 11 studies), while no difference was observed for NSTEMI (RR 0.94, 95% CI 0.81 to 1.08; p=0.38; 6 estimates, 5 studies; figures4  6). For asian versus white patients, there was no significant difference by ACS subtype (STEMI: RR 1.08, 95% CI 0.96 to 1.21; p=0.21; NSTEMI: RR 1.05, 95% CI 0.89 to 1.25; p=0.54; Figures5  6).

**Figure 6:**
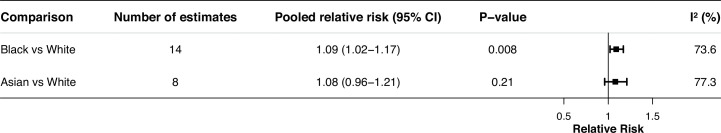
Subgroup analysis of pooled RRs for all-cause mortality following STEMI comparing black versus white and Asian versus white patients. Estimates are presented as RRs with 95% CIs using random-effects models; I² indicates heterogeneity. RRs, relative risks; STEMI, ST-elevation myocardial infarction.

Stratification by study decade for black versus white patients showed no evidence of temporal change in mortality differences across the 1990s (RR 1.03, 95% CI 0.93 to 1.16; p=0.56), 2000s (RR 1.02, 95% CI 0.96 to 1.07; p=0.58) or 2010s (RR 0.96, 95% CI 0.88 to 1.06; p=0.46), with all estimates indicating no statistically significant difference between groups (figure 4). Stratification by study decade for asian versus white patients similarly demonstrated no clear temporal trend in mortality differences, with estimates in the 2000s (RR 1.08, 95% CI 0.93 to 1.24; p=0.32) and 2010s (RR 1.08, 95% CI 0.90 to 1.30; p=0.39; figure 5).

### Publication bias

For black versus white comparisons, visual inspection of the funnel plot suggested some asymmetry (online supplemental figure 3). Egger’s test did not provide statistical evidence of small-study or publication bias (p=0.18). Although the intercept estimate indicated some asymmetry, the lack of statistical significance suggests that this pattern is unlikely to reflect meaningful small-study effects. Consistent with this, trim-and-fill analysis suggested that any potential publication bias had only a limited influence on the overall effect estimate (online supplemental figure 4).

For asian versus white comparisons, interpretation of the funnel plot was limited by the smaller number of studies (online supplemental figure 5). The funnel plot suggested possible asymmetry, but Egger’s test again did not indicate statistically significant publication bias (p=0.16). The intercept estimate was compatible with minor asymmetry, and trim-and-fill analysis indicated that any such bias did not meaningfully alter the pooled effect (online supplemental figure 6).

## Discussion

### Principal findings

In this systematic review and meta-analysis of 14.2 million patients with ACS across 40 studies, we make three key observations. First, overall mortality was similar between black and white patients following ACS, though black patients had significantly higher mortality than white patients specifically following STEMI. Second, overall mortality was similar between asian and white patients across all included studies; however, asian patients demonstrated significantly higher mortality than white patients in US-based studies, while no such difference was observed in UK-based studies. Third, age was a significant effect modifier for black versus white comparisons, with ethnic differences in mortality attenuating in older populations, suggesting that disparities may be most pronounced in younger patients. These findings should be interpreted with caution given the substantial heterogeneity observed across both primary analyses.

Importantly, ethnicity should not be interpreted as a mechanistic determinant of ACS outcomes, but rather as a composite marker intersecting with structural inequity, healthcare access, comorbidity burden and social determinants of health—a framework comprehensively described by Javed *et al*, who apply a social determinants of health lens to racial and ethnic disparities in cardiovascular disease across five structural domains.^15^

### Comparison with other studies

Our pooled estimate showed no overall difference in mortality between black and white patients after ACS. While studies from the USA and UK show that black and asian patients have a higher burden of ACS and adverse cardiovascular risk profiles than white patients, the literature does not conclusively state whether this translates to higher mortality. Our findings align with reports suggesting that in-hospital mortality after ACS may now be similar or even lower in black patients than white patients, despite greater comorbidity and less frequent invasive management.^2 16 17^

The only previous meta-analysis on this topic, by Jaiswal *et al*^8^ reported higher mortality in black than white patients after myocardial infarction but was limited to six older studies with short follow-up and minimal adjustment. Our analysis includes a substantially larger and more contemporary evidence base across the USA, UK and Canada. Importantly, the inclusion of large contemporary registry studies—including a UK-based cohort of over 400 000 patients by Roman *et al*—contributed to a more precise and updated estimate of the black versus white mortality comparison.

Overall, our pooled estimates showed comparable overall mortality in black and white patients. For asian patients, the overall pooled estimate was non-significant; however, significantly higher mortality was observed when analyses were restricted to US-based studies. This is consistent with observations by Patel *et al*,^18^ who highlight distinct cardiovascular risk profiles across South Asian, Chinese and white European populations, noting that excess cardiovascular risk does not manifest uniformly across healthcare settings.

### Disparities in STEMI outcomes between black and white patients

Despite no overall mortality difference between black and white patients after ACS, we observed higher mortality among black patients presenting with STEMI (RR 1.09, 95% CI 1.02 to 1.17). This finding should be interpreted cautiously given that it is based on 11 studies and may be hypothesis-generating rather than definitive; however, it is biologically plausible and consistent with evidence reporting higher mortality among black patients with acute myocardial infarction, often alongside lower rates of coronary angiography and revascularisation.^19^,^23^

A key proposed mechanism relates to symptom presentation. Hravnak *et al* demonstrated that black patients were more likely to report shortness of breath than chest pain, and that shortness of breath was associated with lower rates of revascularisation.^5^ Studies of STEMI care consistently report longer symptom-to-door times, lower use of primary PCI and reduced rates of timely reperfusion among black patients.^1624^,^27^ Osho *et al*, whose data contribute to our STEMI subgroup, showed lower odds of prehospital ECG use and achieving guideline-recommended door-to-device times in black patients.^28^ The absence of a mortality difference for NSTEMI (RR 0.94, 95% CI 0.81 to 1.08) supports the hypothesis that disparities are greatest where treatment timing is critical.

These delays are likely compounded by provider-level influences. Green *et al* demonstrated that unconscious racial bias influenced thrombolysis recommendations for black versus white patients with ACS.^4^

Taken together, atypical symptom presentation, systems-level delays, reduced timely reperfusion and implicit provider bias represent plausible mechanisms underlying greater disparities in STEMI outcomes.

### Disparities in ACS outcomes between Asian and white patients

Overall mortality was similar between asian and white patients across all included studies (RR 1.06, 95% CI 0.95 to 1.17). However, restriction to US-based studies demonstrated significantly higher mortality in asian patients (RR 1.14, 95% CI 1.03 to 1.27), while UK-based studies showed no such difference (RR 0.93, 95% CI 0.83 to 1.04). This geographical variation is clinically important and warrants careful interpretation.

The US-specific finding is consistent with evidence that asian populations in the USA—particularly South Asians—experience disproportionately high coronary artery disease burden, earlier ACS onset and more adverse cardiovascular risk profiles than white patients, including higher rates of type 2 diabetes, central obesity and atherogenic dyslipidaemia at lower body mass index thresholds.^29 30^ The American Heart Association has highlighted that aggregated ‘Asian American’ categories may underestimate South Asian-specific risk, because East Asian populations carry lower cardiovascular risk and that South Asians in the USA may differ from those in other countries in socio-economic status, health behaviours and insurance coverage in ways that affect outcomes.^31^

The null finding in UK-based studies is consistent with prior evidence from Zaman *et al*, who demonstrated using linked national ACS registry data that South Asian patients had better post-ACS prognosis than white patients despite higher disease incidence, suggesting that universal access to care through the National Health Service may mitigate post-ACS mortality disparities.^30^ The contrast between USA and UK findings supports this interpretation, and points to insurance barriers, socio-economic inequalities and differential access to specialist care as potential drivers of the mortality difference observed in US-based Asian patients.

### Sensitivity, subgroup and bias analyses

Sensitivity analyses demonstrated robustness of findings across risk of bias strata, with pooled estimates consistent restricting to low-risk and low-to-moderate-risk studies. There was no evidence of sex-specific differences in post-ACS mortality for black versus white patients, though the estimate for women approached conventional significance thresholds (RR 0.91, 95% CI 0.82 to 1.00; p=0.05), based on only four studies and should be interpreted cautiously. Stratification by study decade showed no significant temporal trend, suggesting that structural contributors to ethnic disparities have persisted despite improvements in overall ACS care.

Age was a significant effect modifier for black versus white comparisons (p=0.0033), with ethnic differences in mortality attenuating in older populations. Several mechanisms may explain this pattern. Insurance-related access barriers may contribute, as black patients under 65 in the USA are disproportionately uninsured or underinsured, delaying care.^2^ Wallace *et al* showed that Medicare eligibility at age 65 reduced black–white insurance disparities and improved access, suggesting that near-universal coverage may attenuate inequities in cardiac care.^32^ Second, survivorship bias may contribute older black patients who survive to present with ACS represent a more selected, physiologically resilient subgroup than their younger counterparts, potentially reducing the apparent ethnic mortality difference in older populations. This is consistent with Kyalwazi *et al*, who found black–white cardiovascular mortality gaps are greatest in younger adults, particularly among women.^33^ Together, these findings suggest that ethnic disparities in ACS mortality disproportionately affect younger black patients, in whom structural barriers to timely care have the most pronounced impact on survival. Age was not a significant modifier for asian versus white comparisons (p=0.48), suggesting different underlying mechanisms.

Assessment of publication bias warrants careful consideration. For black versus white comparisons, visual asymmetry on funnel plot inspection suggested possible small-study effects; however, Egger testing did not reach significance (p=0.18) and the intercept estimate indicated only minor asymmetry. Trim-and-fill analysis suggested that any potential publication bias had only a limited influence on the pooled estimate. For asian versus white comparisons, funnel plot interpretation was limited by the small number of contributing studies, although possible asymmetry was observed. Egger testing (p=0.16) and trim-and-fill analysis suggested no substantial influence of publication bias.

### Strengths and limitations

This study has important strengths. To our knowledge, it represents the most comprehensive synthesis of ethnic differences in post-ACS mortality to date, incorporating 40 studies and 14.2 million patients across the USA, UK and Canada. As death was the primary outcome, outcome misclassification is unlikely. The use of random-effects models, prespecified subgroup analyses, meta-regression and sensitivity analyses by risk of bias all strengthen confidence in the robustness of the findings.

Several limitations warrant consideration. Substantial heterogeneity (I² >90% in both primary analyses) means pooled estimates should be interpreted as the average of a distribution of true effects rather than a single underlying effect. While source of heterogeneity were explored through subgroup and meta-regression analyses, much of this variation remains unexplained and likely reflects genuine differences in populations, healthcare settings, follow-up durations and adjustment strategies across included studies.

Ethnicity definitions varied considerably across included studies. Most did not specify whether ethnic classification was self-reported or administratively assigned, and many used broad categories that may not reflect the populations of interest. The ‘Asian’ category in particular encompasses South Asian, East Asian and Southeast Asian subpopulations with markedly different cardiovascular risk profiles, comorbidity burdens and care pathways. This heterogeneity within the asian category likely attenuates pooled estimates and limits granular interpretation, particularly given that most included studies were conducted in the USA, where ‘Asian’ predominantly captures East Asian populations, whereas in the UK the term more commonly refers to South Asian populations. This cross-national inconsistency complicates pooled analysis and should be explicitly addressed in future registry studies using granular ethnicity data. Smaller sample sizes among ethnic minority groups further limited statistical power for asian-specific analyses. We excluded Hispanic populations because ethnic classifications differ substantially between the USA and UK, limiting meaningful cross-country comparison.

All included studies were observational, and residual confounding related to these factors is likely, meaning observed ethnic differences in mortality should not be interpreted as intrinsic biological effects. Differences in healthcare systems also contributed to heterogeneity—the UK National Health Service provides universal access to care, whereas insurance barriers and greater distances to interventional centres in the USA may exacerbate disparities in access to and quality of care.

Finally, an individual-patient-level meta-analysis would allow more detailed investigation of ethnic classifications, care pathways and outcome differences, and should be a priority for future research in this area.

## Conclusions

In this systematic review and meta-analysis of 14.2 million patients with ACS, overall post-ACS mortality was similar between black and white patients and between asian and white patients. However, black patients had significantly higher mortality than white patients following STEMI, and asian patients demonstrated higher mortality than white patients in US-based studies. The structural and systems-level factors underlying these disparities, including atypical symptom recognition, insurance-related access barriers, delays in reperfusion and implicit provider bias, are most likely to affect younger patients, in whom the survival benefit of timely intervention is greatest. The divergence between USA and UK findings forsian patients underscores that observed ethnic differences in post-ACS mortality are shaped by healthcare system context and the heterogeneity of populations captured under broad ethnic labels rather than fixed biological phenomena. Addressing these disparities will require granular ethnicity data in future registry studies, targeted efforts to improve equitable access to timely reperfusion for black patients presenting with STEMI, and broader structural efforts to reduce socio-economic and insurance-related barriers to cardiac care for younger ethnic minority patients in healthcare systems without universal coverage.

## Supplementary material

10.1136/openhrt-2026-004072 online supplemental table 1

10.1136/openhrt-2026-004072 online supplemental file 1

## Data Availability

Data are available in a public, open access repository.
