# Supplementary material for: Ethnic disparities in mortality from acute coronary syndromes: a systematic review and meta-analysis
Source: Open Heart. 2026 Apr 30;13(1):e004072. doi: 10.1136/openhrt-2026-004072 (PMC13141094; doi:10.1136/openhrt-2026-004072)
Supplement: online supplemental table 1 [file openhrt-13-1-s001.pdf]

Supplementary Table 1: Study Characteristics

Study	Population	Sample size	Male (%)	Study type	Region	Period	Mean age (years)	Exposure (ethnicity)	Mortality follow-up	Data source
Bhatia et al (2023)	STEMI	96,669	62.8	RCS	US	1994–2015	64.5	Black	1-year	Linked Hospital Data
Rymer et al (2022)	STEMI	816,894	65.0	RCS	US	2008–2017	69.5	Black	1-year	Linked Hospital Data
Bolorundoro et al (2016)	STEMI	1,144	66.3	RCS	US	2008–2013	58.0	Black	1-year	EHR (single centre)
Singh et al (2014)	AMI	4,045,267	49.6	RBA	UK	1992–2010	N/A	Black	30-day	Medicare Claims
Echols et al (2007)	NSTEMI	6,663	64.9	SA-RCT	US	2001–2003	66.5	Black	1-year	Clinical Trial Data
Petersen et al (2002)	AMI	4,611	100.0	RCS	US	1994–1995	65.8	Black	30-day	EHR (multicentre)
Mickelson et al (1997)	AMI	316	100.0	RCS	US	1993–1995	64.5	Black	22-month	EHR (single centre)
Matthews et al (2014)	ACS	40,500	54.7	RCS	US	2001–2006	77.0	Black	1-year	Linked Hospital Data
Barnato et al (2005)	ACS	138,995	53.6	RCS	US	1994–1995	75.0	Black	1-year	Linked Hospital Data
Ford et al (2000)	AMI	10,016	53.3	RCS	US	1994–1995	76.0	Black	30-day	Clinical Registry Data
Taylor et al (1997)	AMI	1,263	73.7	RBA	US	1993	60.4	Black	30-day	Hospital Chart Data
Patel et al (2023)	STEMI	3,990	68.0	RCS	US	2020	66.3	Black/Asian	In-hospital	Administrative Claims
Ashraf et al (2023)	AMI	7,009,536	58.2	RCS	US	2005–2017	67.0	Black	In-hospital	Administrative Claims
Yong et al (2018)	ACS	565,791	58.9	RCS	US	2005–2014	67.0	Black/Asian	In-hospital	Administrative Claims
Brown et al (2016)	STEMI	768	75.3	RCS	UK	2008–2013	58.7	Black	In-hospital	EHR (single centre)
Anstey et al (2016)	STEMI	69,500	69.7	RCS	US	2004–2011	57.0	Black	In-hospital	National Registry
Casale et al (2007)	AMI	15,854	63.6	RCS	US	2003	N/A	Black	In-hospital	Administrative Claims
Mitchell et al (1995)	AMI	18,202	50.9	RCS	US	1992	73.8	Black	90-day	Administrative Claims
Sonei et al (2005)	NSTEMI	43,317	59.4	RBA	US	2002–2005	65.5	Black	In-hospital	Hospital Chart Data
Popescu et al (2007)	AMI ± revascularisation	1,215,924	48.0	RCS	US	2000–2005	79.1	Black	In-hospital	Administrative Claims
Mehta et al (2006)	STEMI	32,419	72.3	SA-RCT	US	1990–2000	60.9	Black	1-year	Pooled Clinical Trials
Graham et al (2018)	AMI	6,402	66.8	PCS	US	2003–2008	60.0	Black	5-year	Acute MI Registry
Blackston et al (2020)	AMI	1,122	54.6	PCS	US	2003–2016	73.2	Black	3-year	Primary Cohort Data
Iribarren et al (2005)	AMI	18925	100	RCS	US	1993-2002	63.4	Black	8-year	EHR (multicentre)
Kaila et al (2014)	AMI	69,908	70.7	RCS	Canada	1999-2012	60.3	Asian	1-year	Clinical Registry Data
Kawsara et al (2022)	STEMI	332,620	69.3	RCS	US	2016-2019	63.5	Asian	In-hospital	Administrative Claims
Kim et al (2018)	AMI	438,936	60.0	RCS	US	2010–2011	68.3	Black/Asian	In-hospital	Administrative Claims
Maynard et al (1997)	AMI	11,254	64.9	RCS	US	1988–1994	65.8	Black	7-year	Clinical Registry Data
Spertus et al (2005)	AMI	1,159	61.4	RCS	US	2000–2001	61.7	Black	1-year	Clinical Registry Data
Srivastava et al (2022)	AMI	1,734,448	61.6	RCS	US	2015–2018	67.1	Black	In-hospital	Administrative Claims
Wang et al (2007)	NSTEMI	73,584	61.4	RCS	US	2005	69.0	Asian	In-hospital	Clinical Registry Data
Roman et al (2025)	AMI	428,700	63.6	RCS	UK	2015–2018	70.0	Black/Asian	4.5-year	Linked Hospital Data
Osho et al (2023)	STEMI	178,062	70.6	RCS	US	2015–2021	62.0	Black	In-hospital	Clinical Registry Data
Thyagaturu et al (2024)	AMI	820,893	61.9	RCS	US	2020	67.7	Black	In-hospital	Administrative Claims
Vijay et al (2024)	AMI	146,962	66.8	RCS	US	2015–2021	66.5	Asian	In-hospital	Clinical Registry Data
Hammershaimb et al (2022)	AMI	1,047	84.1	RCS	US	2006–2016	44.3	Black/Asian	7.5-year	EHR (multicentre)
Kumaran et al (2023)	STEMI	298,223	100	RCS	US	2016-2019	68.5	Black/Asian	In-hospital	Administrative Claims
Arora et al (2017)	NSTEMI	17,755	59.7	RCS	US	2000–2014	63	Black	1-year	Clinical Registry Data
Gholap et al (2015)	AMI	4,111	70.2	RCS	UK	2002–2008	66.4	Asian	3-year	Clinical Registry Data
Ijaz et al (2023)	AMI	1,682,963	44.1	RCS	US	2004–2018	85.8	Black	In-hospital	Administrative Claims

Abbreviations: acute coronary syndrome (ACS); acute myocardial infarction (AMI); ST-segment elevation myocardial infarction (STEMI); non–ST-segment elevation myocardial infarction (NSTEMI); retrospective cohort study (RCS); retrospective cohort analysis (RBA); prospective cohort study (PCS); subgroup analysis of a randomised controlled trial (SA-RCT); electronic health record (EHR); United States (US); United Kingdom (UK)
