# Supplementary material for: Ethnic disparities in mortality from acute coronary syndromes: a systematic review and meta-analysis
Source: Open Heart. 2026 Apr 30;13(1):e004072. doi: 10.1136/openhrt-2026-004072 (PMC13141094; doi:10.1136/openhrt-2026-004072)
Supplement: online supplemental file 1 [file openhrt-13-1-s002.docx]

**Supplementary Methods**

*Search Strategy*

We performed a comprehensive search across four databases, including Embase, Global Health, Ovid MEDLINE and Web of Science. Web of Science requires slightly different formatting. The same search was run but formatted appropriately for each database.

Embase, Global Health, Ovid MEDLINE

((Invasive Strategy or Conservative Strategy or Invasive Management or Conservative Management or Invasive Treatment or Conservative Treatment or Invasive Therapy or Conservative Therapy

or "Early invasive strategy versus ischemia-guided strategy" or "Early invasive strategy versus ischemia-guided strategies" or "Early invasive strategy versus ischaemia-guided strategies"

or "Interventional versus conservative treatment" or "Invasive versus non-invasive strategy" or "Early Invasive Versus Selective Strategy"

or "Invasive versus non-invasive treatment" or "Percutaneous Coronary Angioplasty" or "Coronary Angioplasty" or PTCA

or "Conservative Strategy" or "Conservative Management" or "Conservative Treatment" or "Conservative Therapy"

or "Optimal Medical Strategy" or "Optimal Medical Treatment" or "Optimal Medical Management" or "Optimal Medical Therapy"

or "Routine Early Invasive Management versus Conservative Management"

or balloon angioplast* or percutaneous coronary intervention* or "intervention* percutaneous coronary"

or "revascularization* percutaneous coronary" or "angioplast* coronary" or "percutaneous coronary"

or "revascularization" or "transluminal coronary"))

AND

((acute coronary syndrome* or ACS or unstable angina* or UA or NSTEMI or STEMI or non-STEMI

or "non-ST elevation acute coronary syndrome" or NSTEACS or "non-ST elevation myocardial infarction"

or "non-Q wave MI" or "non-Q wave myocardial infarction" or "acute coronary syndrome"

or "Q-wave MI" or "Q-wave myocardial infarction" or "ST elevat* myocardial infarction*"

or "non-ST elevat* myocardial infarction*" or "non-ST-segment elevation ACS" or NSTE-ACS

or myocardial infarct* or myocardial dysfunction or heart attack* or heart infarct*

or angina or stenocardia or coronary thrombosis or acute coronary

or heart disease* or coronary disease* or IHD or CIHD or CHD

or (myocardial adj2 (infarction* or ischaemia*)) or (unstable adj2 angina) or ((preinfarct* or pre infarct*) adj2 angina)

or (("myocardial" or "myocardium" or "subendocardial" or "transmural" or "cardiac" or "cardial" or "coronary" or "heart")

adj2 ("infarct*" or "postinfarct*" or "hypoxi*" or "anoxi*" or "failure*" or "decompensation" or "insufficien*"))

or (("ischemi*" or "ischaemi*")

adj2 ("myocardium" or "myocardial" or "heart" or "coronary" or "cardiac" or "cardial" or "subendocardial" or "cardiomyopath*"))

or (("artery occlusion*" or "artery disease*" or "arterioscleros*" or "atheroscleros*") adj2 coronary)))

AND

(("major adverse cardiovascular events" or MACE or "all-cause mortality" or mortality or death

or "cardiovascular mortality" or "cardiovascular death" or "cardiac death"

or "myocardial infarction" or MI or heart attack

or stroke or "cerebrovascular disease" or "cerebrovascular accident" or "cerebrovascular event"

or "cerebral infarct" or "cerebral hemorrhage" or "cerebral bleed"

or "cvd infarct" or "cvd bleed" or "cvd hemorrhage"

or "recurrent angina" or "recurrent chest pain"))

AND

((ethnicity or ethnic or "group, minority" or "ethnic group" or "population groups" or "continental population groups"

or "Hispanic Americans" or "African Continental Ancestry Group" or "American Native Continental Ancestry Group"

or "Asian Continental Ancestry Group" or "European Continental Ancestry Group" or "Oceanic Ancestry Group"

or "African Americans" or Arabs or "Americans, Asian"

or multicultural or multi cultural or crosscultural or cross cultural or transcultural or trans cultural

or BAME or minority or minorities or ethnology

or "black people" or "white people" or "asian people" or "ethnic groups"

or "black or african american" or "east asian people" or "black person"

or "caucasian race" or "southeast asian people")

or

(ethnicity or "ethnic background" or "ethnocultural background" or ancestry or heritage or descent or nationality or origin

or "cultural heritage" or "ethnic origin" or "racial background" or "racial identity" or "racial heritage" or "racial descent"

or "racial group" or "racial category"

or Black or "British African" or Caribbean or "African American"

or Asian or Indian or Pakistani or Bangladeshi or Chinese

or White or "African descent" or "Afro-Caribbean" or "African diaspora" or "African heritage"

or "People of African origin" or "Black British" or "Afro-British"

or "African diaspora in Britain" or "British people of African descent"

or "West Indian" or "Caribbean diaspora" or "Caribbean heritage" or "People of Caribbean origin"

or "Black American" or "African descent in the United States" or "Afro-American"

or "African diaspora in America" or "People of African origin in the United States"

or "East Asian" or "South Asian" or "Southeast Asian"

or "Asian diaspora" or "People of Asian origin"

or "Indo-Caribbean" or "Indian diaspora" or "People of Indian origin"

or "Pakistani diaspora" or "People of Pakistani origin"

or "Bangladeshi diaspora" or "People of Bangladeshi origin"

or "Chinese diaspora" or "People of Chinese origin"

or Caucasian or "European descent" or "European heritage" or "People of European origin"))

Limits: English, Humans

Web of Science

(

(Invasive Strategy OR Conservative Strategy OR Invasive Management OR Conservative Management OR Invasive Treatment OR Conservative Treatment OR Invasive Therapy OR Conservative Therapy

OR "Early invasive strategy versus ischemia-guided strategy" OR "Early invasive strategy versus ischemia-guided strategies" OR "Early invasive strategy versus ischaemia-guided strategies"

OR "Interventional versus conservative treatment" OR "Invasive versus non-invasive strategy" OR "Early Invasive Versus Selective Strategy"

OR "Invasive versus non-invasive treatment" OR "Percutaneous Coronary Angioplasty" OR "Coronary Angioplasty" OR PTCA

OR "Conservative Strategy" OR "Conservative Management" OR "Conservative Treatment" OR "Conservative Therapy"

OR "Optimal Medical Strategy" OR "Optimal Medical Treatment" OR "Optimal Medical Management" OR "Optimal Medical Therapy"

OR "Routine Early Invasive Management versus Conservative Management"

OR balloon angioplast* OR percutaneous coronary intervention* OR "intervention* percutaneous coronary"

OR "revascularization* percutaneous coronary" OR "angioplast* coronary" OR "percutaneous coronary"

OR "revascularization*" OR "transluminal coronary")

)

AND

(

(acute coronary syndrome* OR ACS OR unstable angina* OR UA

OR (myocardial NEAR/2 (infarction* OR ischaemia* OR ischemia*))

OR heart attack* OR NSTEMI OR STEMI OR non-STEMI

OR "non-ST elevation acute coronary syndrome" OR NSTEACS

OR "non-ST elevation myocardial infarction"

OR "non-Q wave MI" OR "non-Q wave myocardial infarction"

OR "acute coronary syndrome"

OR "Q-wave MI" OR "Q-wave myocardial infarction"

OR (unstable NEAR/2 angina)

OR ((preinfarct* OR "pre infarct*") NEAR/2 angina)

OR "ST elevat* myocardial infarction*"

OR "non-ST elevat* myocardial infarction*"

OR "non-ST-segment elevation ACS" OR NSTE-ACS

OR myocardial dysfunction OR angina OR stenocardia

OR coronary thrombosis OR acute coronary

OR heart disease* OR coronary disease* OR IHD OR CIHD OR CHD

OR (("myocardial" OR "myocardium" OR "subendocardial" OR "transmural"

OR "cardiac" OR "cardial" OR "coronary" OR "heart")

NEAR/2 ("infarct*" OR "postinfarct*" OR "hypoxi*" OR "anoxi*"

OR "failure*" OR "decompensation" OR "insufficien*"))

OR (("ischemi*" OR "ischaemi*")

NEAR/2 ("myocardium" OR "myocardial" OR "heart" OR "coronary"

OR "cardiac" OR "cardial" OR "subendocardial" OR "cardiomyopath*"))

OR (("artery occlusion*" OR "artery disease*" OR "arterioscleros*"

OR "atheroscleros*") NEAR/2 coronary)

OR myocardial infarct* OR heart infarct*)

)

AND

(

("major adverse cardiovascular events" OR MACE OR "all-cause mortality" OR mortality OR death

OR "cardiovascular mortality" OR "cardiovascular death" OR "cardiac death"

OR "myocardial infarction" OR MI OR heart attack

OR stroke OR "cerebrovascular disease" OR "cerebrovascular accident" OR "cerebrovascular event"

OR "cerebral infarct" OR "cerebral hemorrhage" OR "cerebral bleed"

OR "cvd infarct" OR "cvd bleed" OR "cvd hemorrhage"

OR "recurrent angina" OR "recurrent chest pain")

)

AND

(

(ethnicity OR ethnic OR "group, minority" OR "ethnic group" OR "population groups" OR "continental population groups"

OR "Hispanic Americans" OR "African Continental Ancestry Group" OR "American Native Continental Ancestry Group"

OR "Asian Continental Ancestry Group" OR "European Continental Ancestry Group" OR "Oceanic Ancestry Group"

OR "African Americans" OR Arabs OR "Americans, Asian"

OR multicultural OR "multi cultural" OR crosscultural OR "cross cultural" OR transcultural OR "trans cultural"

OR BAME OR minority OR minorities OR ethnology

OR "black people" OR "white people" OR "asian people" OR "ethnic groups"

OR "black or african american" OR "east asian people" OR "black person"

OR "caucasian race" OR "southeast asian people"

OR ethnicity OR "ethnic background" OR "ethnocultural background"

OR ancestry OR heritage OR descent OR nationality OR origin

OR "cultural heritage" OR "ethnic origin"

OR "racial background" OR "racial identity" OR "racial heritage" OR "racial descent"

OR "racial group" OR "racial category"

OR Black OR "British African" OR Caribbean OR "African American"

OR Asian OR Indian OR Pakistani OR Bangladeshi OR Chinese

OR White OR "African descent" OR "Afro-Caribbean"

OR "African diaspora" OR "African heritage"

OR "People of African origin"

OR "Black British" OR "Afro-British"

OR "African diaspora in Britain"

OR "British people of African descent"

OR "West Indian"

OR "Caribbean diaspora" OR "Caribbean heritage" OR "People of Caribbean origin"

OR "Black American"

OR "African descent in the United States" OR "Afro-American"

OR "African diaspora in America" OR "People of African origin in the United States"

OR "East Asian" OR "South Asian" OR "Southeast Asian"

OR "Asian diaspora" OR "People of Asian origin"

OR "Indo-Caribbean" OR "Indian diaspora" OR "People of Indian origin"

OR "Pakistani diaspora" OR "People of Pakistani origin"

OR "Bangladeshi diaspora" OR "People of Bangladeshi origin"

OR "Chinese diaspora" OR "People of Chinese origin"

OR Caucasian OR "European descent" OR "European heritage" OR "People of European origin")

)

Filters applied

- English language
- Document type: Article
- Research areas: Cardiovascular System, Cardiology and General Internal Medicine

*Data Extraction*In Iribarren, 2005, outcomes were reported as Black/Asian women vs White men and Black/Asian men vs White men. To ensure comparability with other included studies using ethnically matched reference groups, only the male comparison (Black men vs White men) was included. Female data were excluded due to non-equivalent comparator groups, which could introduce confounding by sex.

Back Calculation

Some of the included studies in our analysis only provided the proportion of patients that died after Acute Coronary Syndrome (ACS) with or without a corresponding p-value, they did not provide the relative risk (RR). Or they provided an effect estimate and p-value but no 95% confidence interval. These studies were Rymer, Bolorundo, Singh, Mickelson, Kaila, Kawsara, Srivastava, and Spertus.

We back calculated the RR and 95% Confidence Intervals (CI) using the following method: e.g. In the Mickelson *et al* 1997 paper

Black patients: 26 deaths out of 84

White patients: 54 deaths out of 232

Calculated risk in each group

Black risk = 26 / 84 = 0.3095

White risk = 54 / 232 = 0.2328

Calculate relative risk

0.3095 / 0.2328 = 1.33

Calculate Standard Error of log(RR)

**
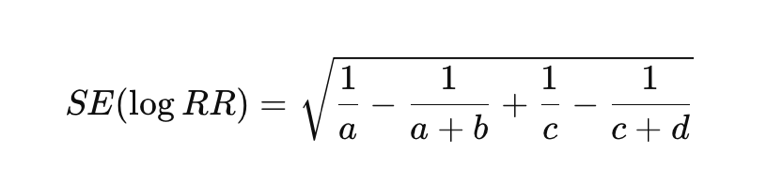
**

a = 26 (Black deaths)

b = 58 (Black survivors)

c = 54 (White deaths)

d = 178 (White survivors)


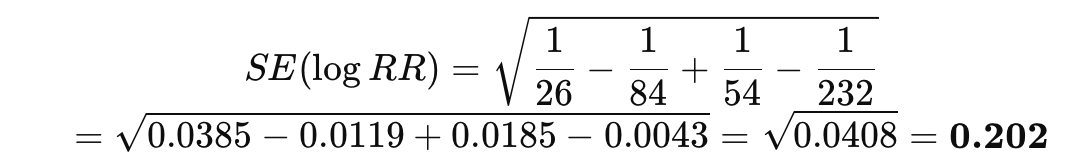


Compute 95% CI for log(RR)


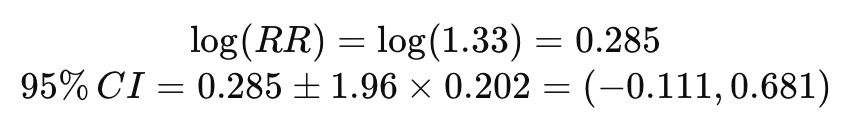


Convert back to RR scale


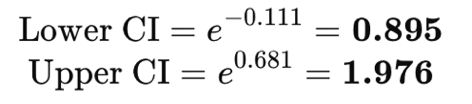


Results

RR (Black vs White) = 1.33 (0.90 – 1.98)

*Supplementary Table 2: Exclusion Criteria*

| **Domain** | *Supplementary appendix 2* | **Exclusion criteria** |
| --- | --- | --- |
| Study design | *Supplementary appendix 2* | Randomised controlled trials without ethnic breakdown of outcomes; case reports; case series; narrative reviews; editorials; letters without primary data; conference abstracts without full text; non-human studies. |
| Population | *Supplementary appendix 2* | Paediatric populations; papers that only included patients who had undergone revascularisation; patients with stable coronary artery disease only; mixed cardiovascular populations where acute coronary syndrome outcomes cannot be separated e.g. with cardiac arrest, cardiogenic shock, stroke, left ventricular aneurysm |
| Exposure / ethnicity | *Supplementary appendix 2* | Studies that do not report ethnicity; studies that do not stratify outcomes by ethnic group; studies using only broad categories (for example ‘minority’ vs ‘non-minority’) that cannot be mapped to predefined groups. |
| Comparator | *Supplementary appendix 2* | Studies without a White or White European comparator group. |
| Outcomes | *Supplementary appendix 2* | Studies reporting only process measures (for example, procedures, investigations) without clinical outcomes; studies with non-extractable or insufficient outcome data. |
| Setting and timeframe | *Supplementary appendix 2* | Non-clinical settings; non-English language publications; duplicate publications from the same cohort where a more complete or recent report is available. |

*Supplementary Table 3 – Variables Adjusted for in Each Study*

*Abbreviations*
- PCI = Percutaneous Coronary Intervention, CABG = Coronary Artery Bypass Graft

*Footnotes*

- “1” = the study adjusted is for the variable; “0” = no adjustment
- 2+ Comorbidities Adjusted = adjustment for at least two comorbidities (e.g., diabetes, hypertension, hyperlipidaemia)
- Angiogram Adjusted = adjustment for receipt of coronary angiography
- PCI Adjusted = adjustment for receipt of PCI
- CABG Adjusted = adjustment for receipt of CABG


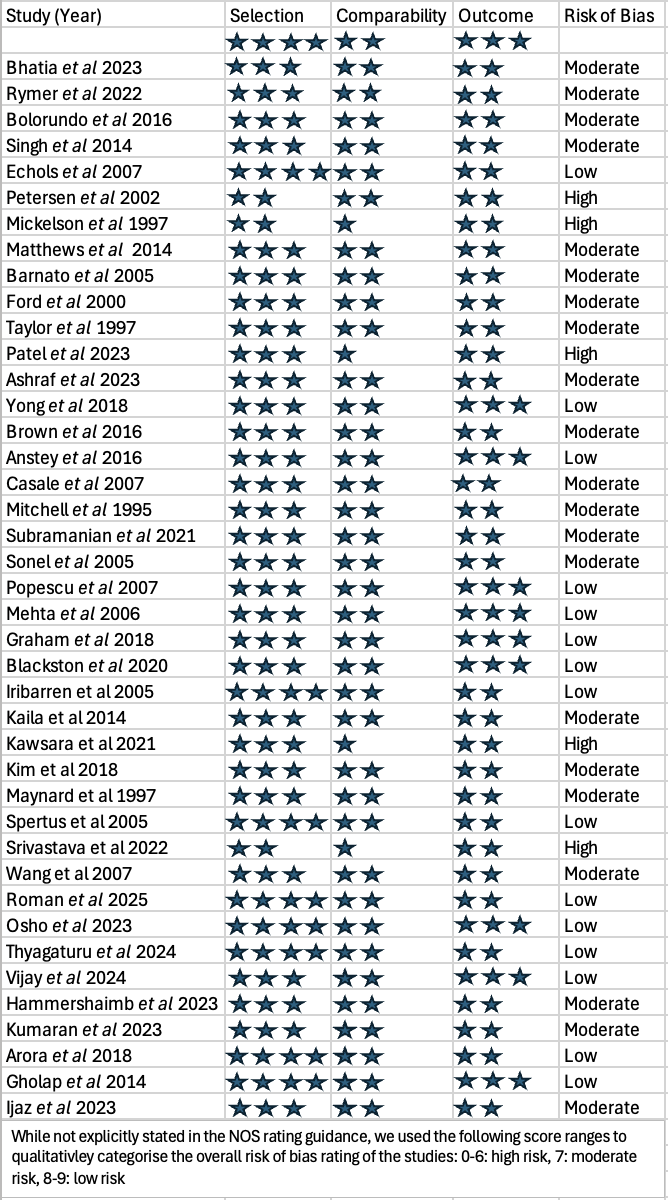
*Supplementary Table 4 – Quality of included studies as assessed by the Newcastle - Ottawa (NOS) quality assessment scale*

Abbreviations

- NOS = Newcastle–Ottawa Scale
- ACS = Acute Coronary Syndrome
- AMI = Acute Myocardial Infarction

STEMI = ST-elevation myocardial infarction

NSTEMI = Non-ST-elevation myocardial infarction

**Supplementary Figures**

*Supplementary Figure 1 – Ethnic Groups versus White patients: mortality after ACS*


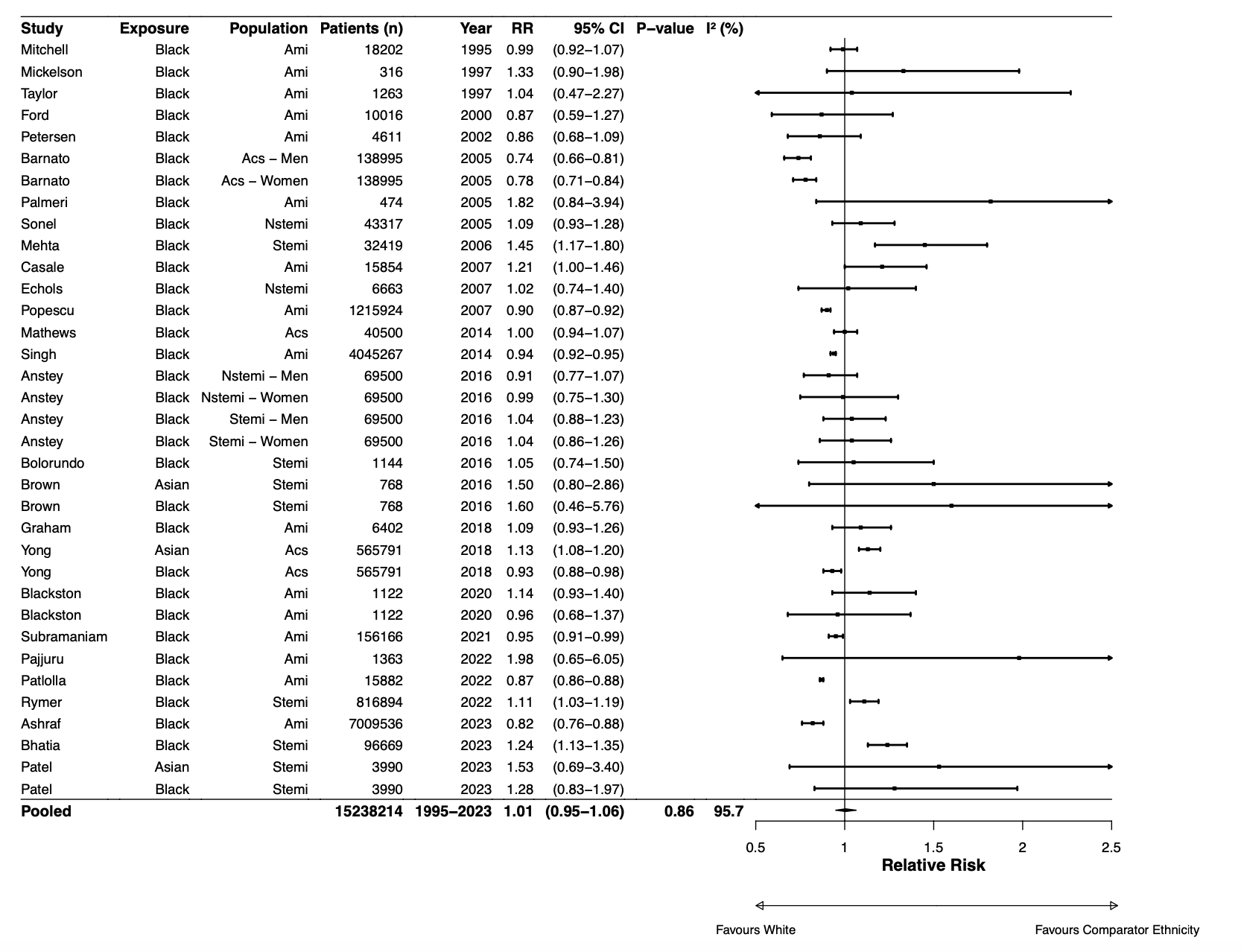


Comparator: Ethnic minority patients; Reference group: White patients
Abbreviations: ACS, acute coronary syndrome; AMI, acute myocardial infarction; STEMI, ST-elevation myocardial infarction; NSTEMI, non–ST-elevation myocardial infarction; RR, relative risk; CI, confidence interval; I², heterogeneity statistic.


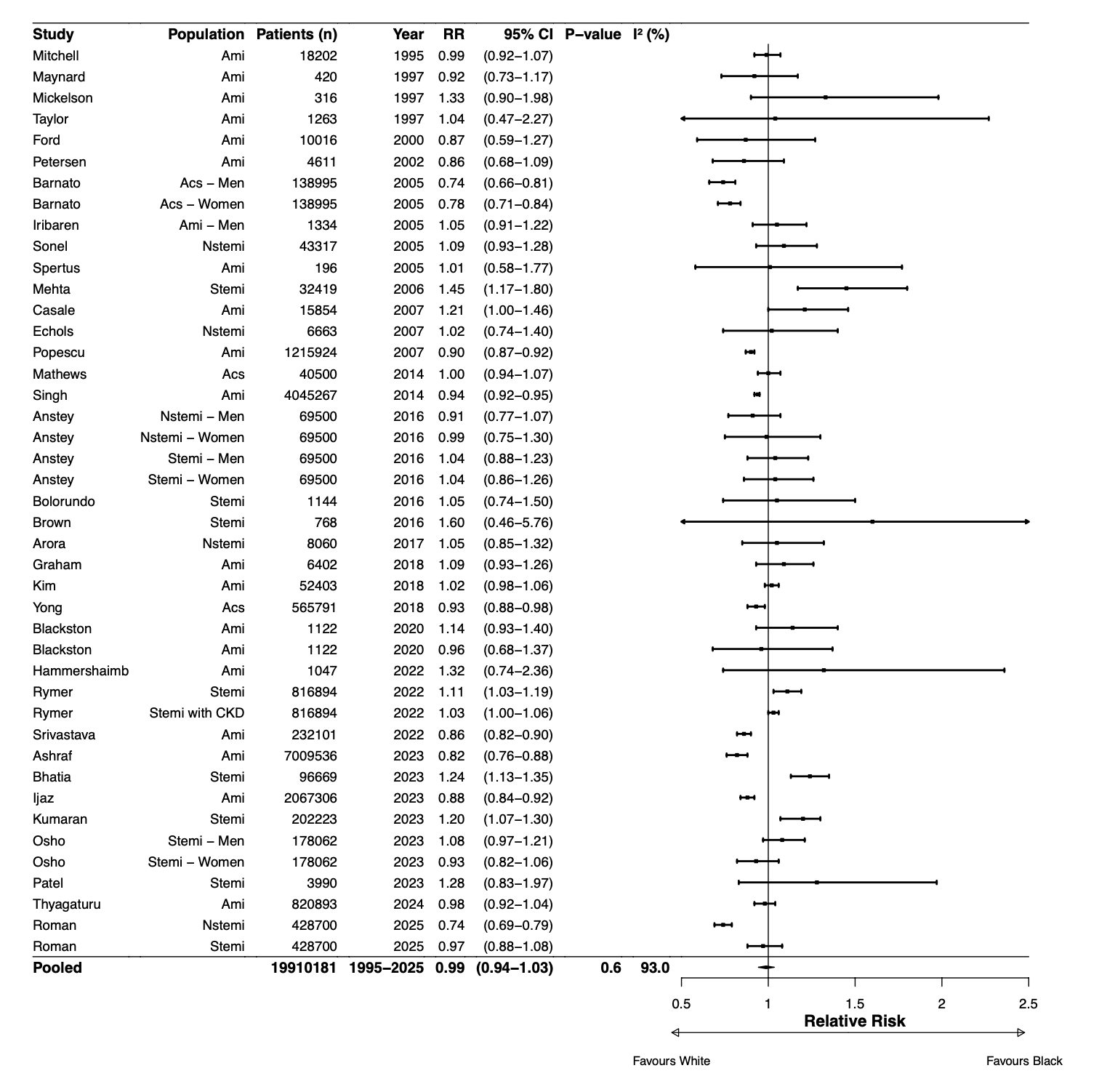
*Supplementary Figure 2 – Black patients versus White patients: mortality after ACS*

Comparator: Black patients; Reference group: White patients
Abbreviations: ACS, acute coronary syndrome; AMI, acute myocardial infarction; STEMI, ST-elevation myocardial infarction; NSTEMI, non–ST-elevation myocardial infarction; RR, relative risk; CI, confidence interval; I², heterogeneity statistic.

*Supplementary Figure 3 – Asian patients versus White patients: mortality after ACS*


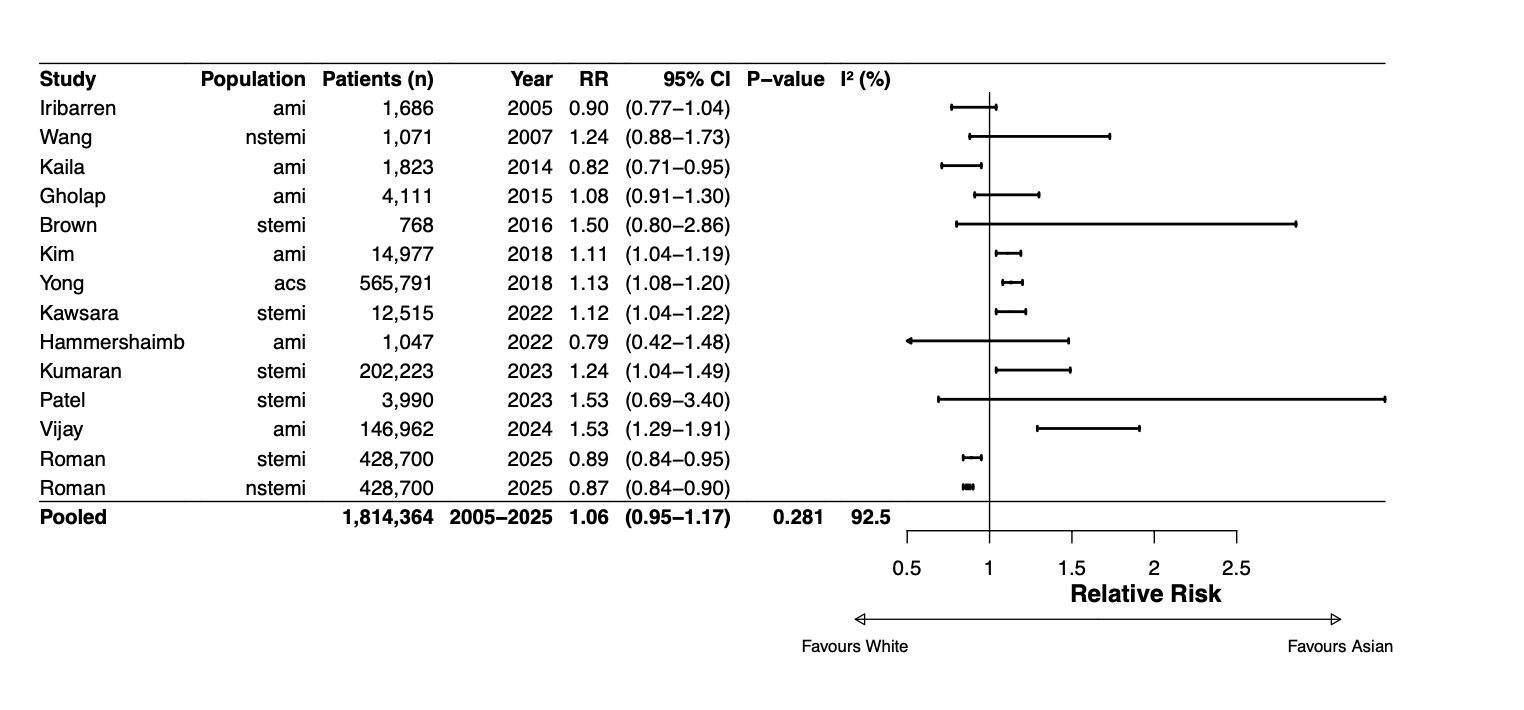


Comparator: Asian patients; Reference group: White patients
Abbreviations: ACS, acute coronary syndrome; AMI, acute myocardial infarction; STEMI, ST-elevation myocardial infarction; NSTEMI, non–ST-elevation myocardial infarction; RR, relative risk; CI, confidence interval; I², heterogeneity statistic.

*Supplementary Figure 4 – Funnel Plot for studies with Black versus White patients*


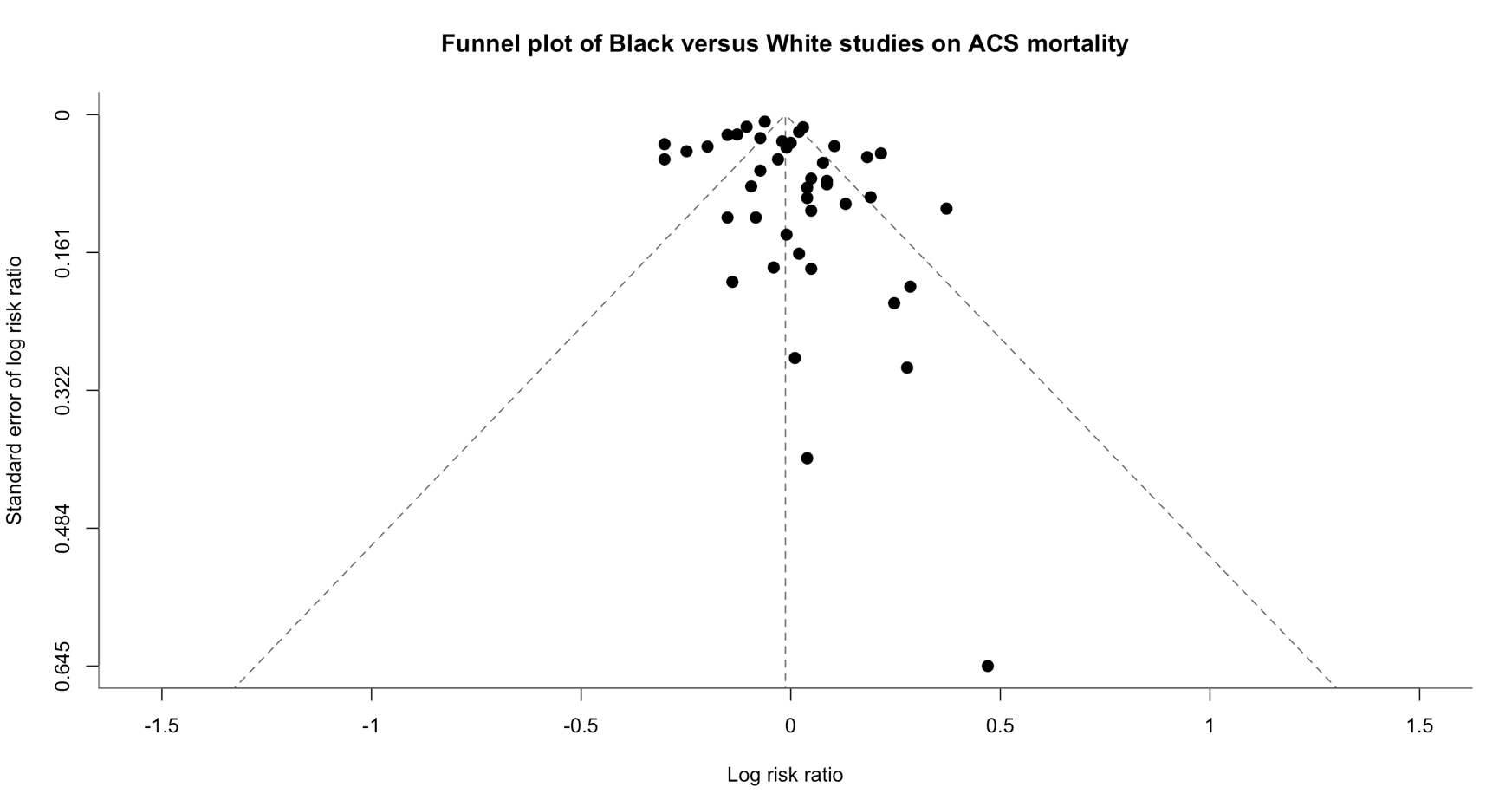


Abbreviations:

- ACS = Acute Coronary Syndrome
- SE = Standard Error
- RR = Relative Risk

Footnotes:

- The x-axis shows the log risk ratio for mortality comparing ethnic groups with White patients
- The y-axis shows the standard error of the log risk ratio, studies with larger sample sizes appear higher on the plot, while smaller studies appear lower
- The vertical dashed line represents the pooled effect estimate
- The diagonal dashed lines represent the expected 95% confidence region in the absence of publication bias
- The funnel plot shows some visual asymmetry, although formal statistical testing did not demonstrate significant small-study effects or publication bias.

*Supplementary Figure 5 – Duval and Tweedie’s Trim and Fill Method for studies with Black versus White patients*


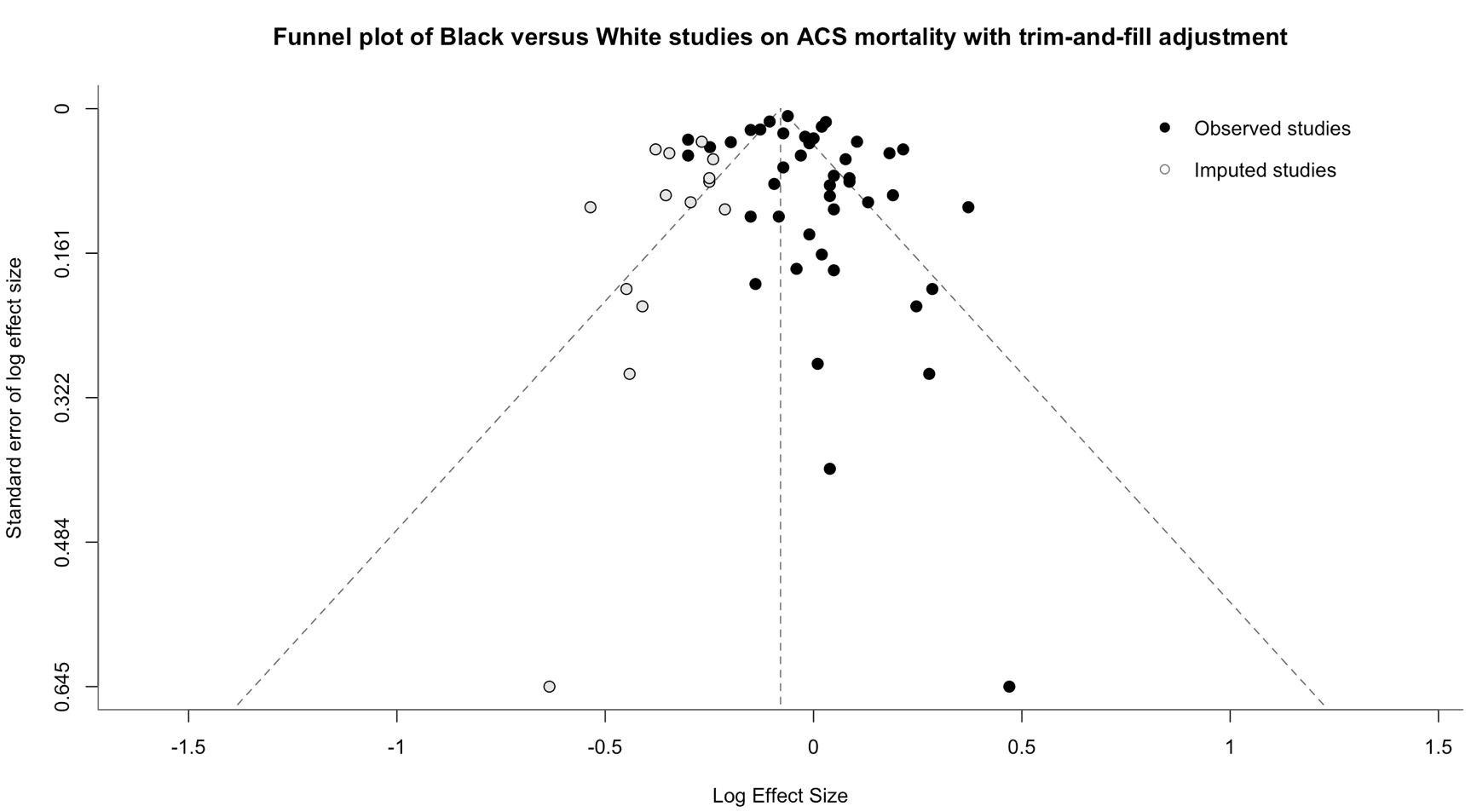


Abbreviations:

- ACS = Acute Coronary Syndrome
- SE = Standard Error
- RR = Relative Risk

Footnotes:

- Black dots represent *observed studies* included in the meta-analysis.
- Empty dots represent *imputed studies* added by the trim-and-fill method to adjust for asymmetry
- The x-axis shows the log effect size for mortality comparing ethnic groups with White patients
- The y-axis shows the standard error of the log effect size; larger studies appear higher on the plot, smaller studies lower
- This funnel plot shows some asymmetry, with trim-and-fill identifying potentially missing studies on the left; however, the adjusted analysis suggests that any publication bias has a limited influence on the overall effect estimate.

*Supplementary Figure 6 – Funnel Plot for studies with Asian versus White patients*

*
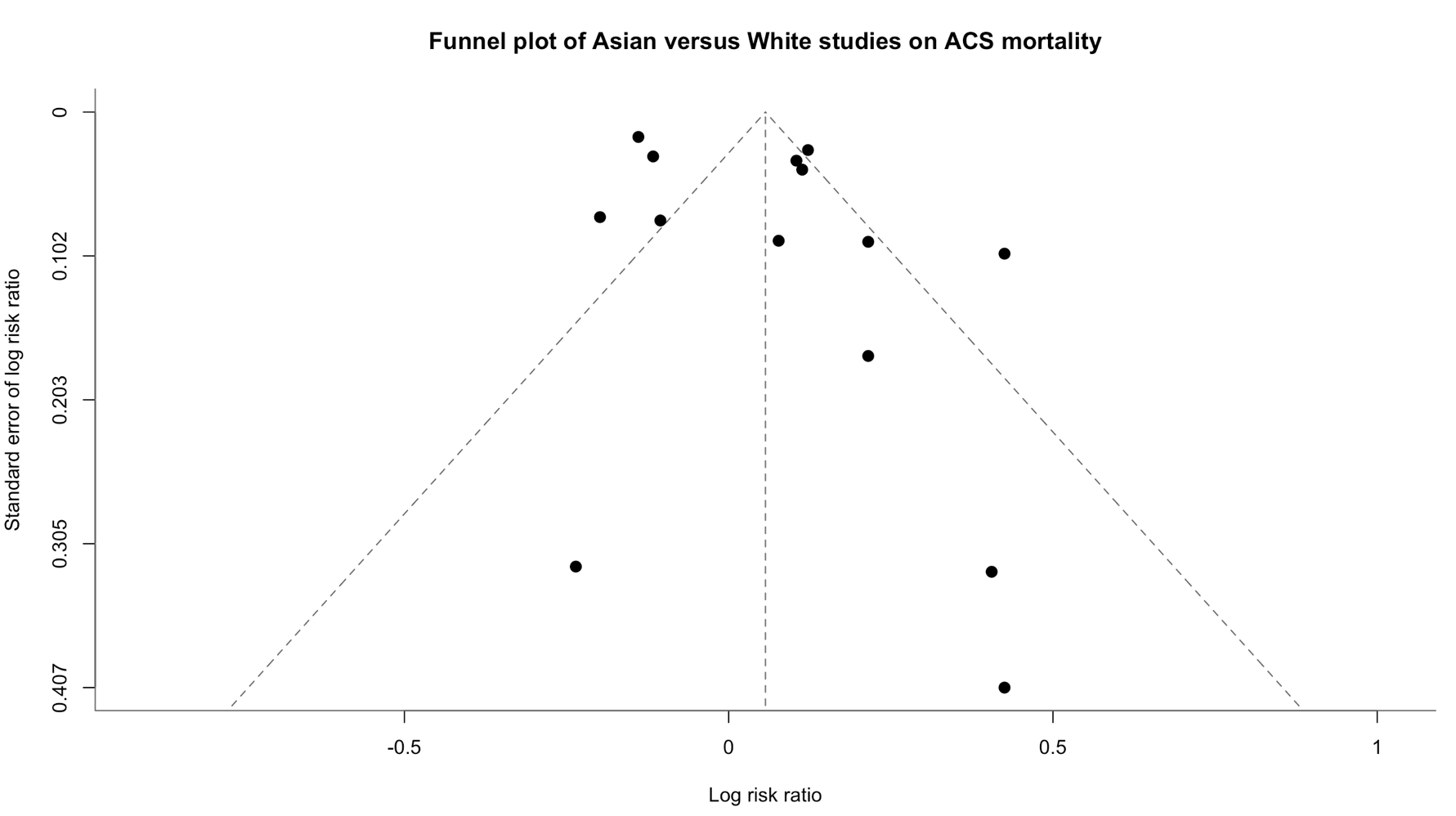
*

Abbreviations:

- ACS = Acute Coronary Syndrome

- SE = Standard Error

- RR = Relative Risk

Footnotes:

- The x-axis shows the log risk ratio for mortality comparing Asian and White patients.
- The y-axis shows the standard error of the log risk ratio; studies with larger sample sizes (greater precision) appear toward the top of the plot, while smaller studies appear toward the bottom.
- The vertical dashed line represents the pooled effect estimate.
- The diagonal dashed lines represent the expected 95% confidence region in the absence of publication bias.
- Interpretation of the funnel plot is limited by the smaller number of studies; although some visual asymmetry is present, formal statistical testing did not demonstrate significant small-study effects or publication bias.

*Supplementary Figure 7 – Duval and Tweedie’s Trim and Fill Method for studies with Asian versus White patients*

*
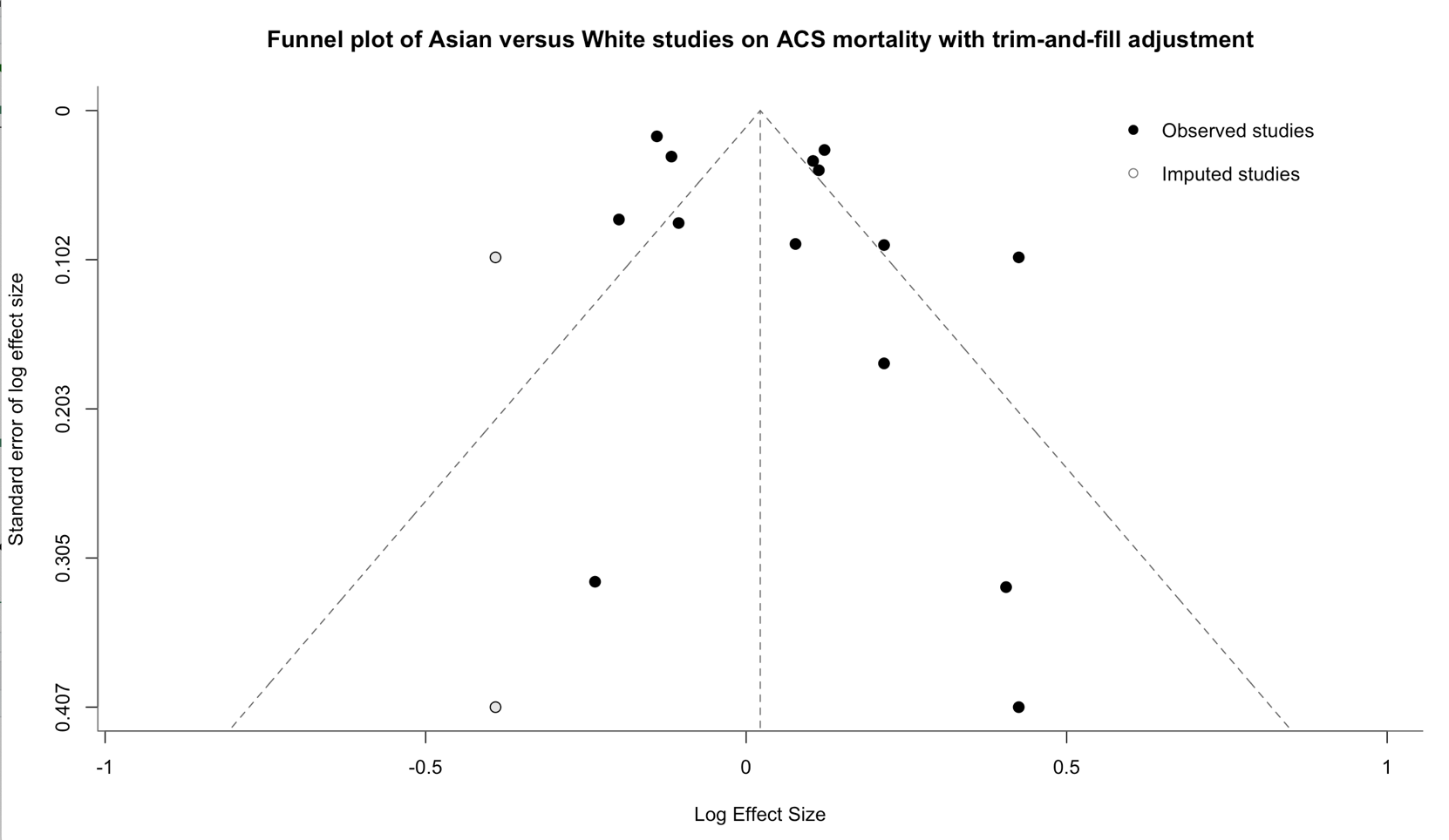
*

Abbreviations

- ACS = Acute Coronary Syndrome
- SE = Standard Error
- RR = Relative Risk

Footnotes

- Black dots represent observed studies included in the meta-analysis.
- White (empty) dots represent studies imputed using the trim-and-fill method to account for potential publication bias.
- The x-axis shows the log effect size for mortality comparing Asian and White patients.
- The y-axis shows the standard error of the log effect size, with more precise (larger) studies appearing toward the top and less precise (smaller) studies toward the bottom.
- The funnel plot demonstrates mild asymmetry, with trim-and-fill suggesting potentially missing studies on the left-hand side. However, the adjusted estimate indicates that any small-study effects or publication bias are unlikely to meaningfully alter the overall effect.
- *
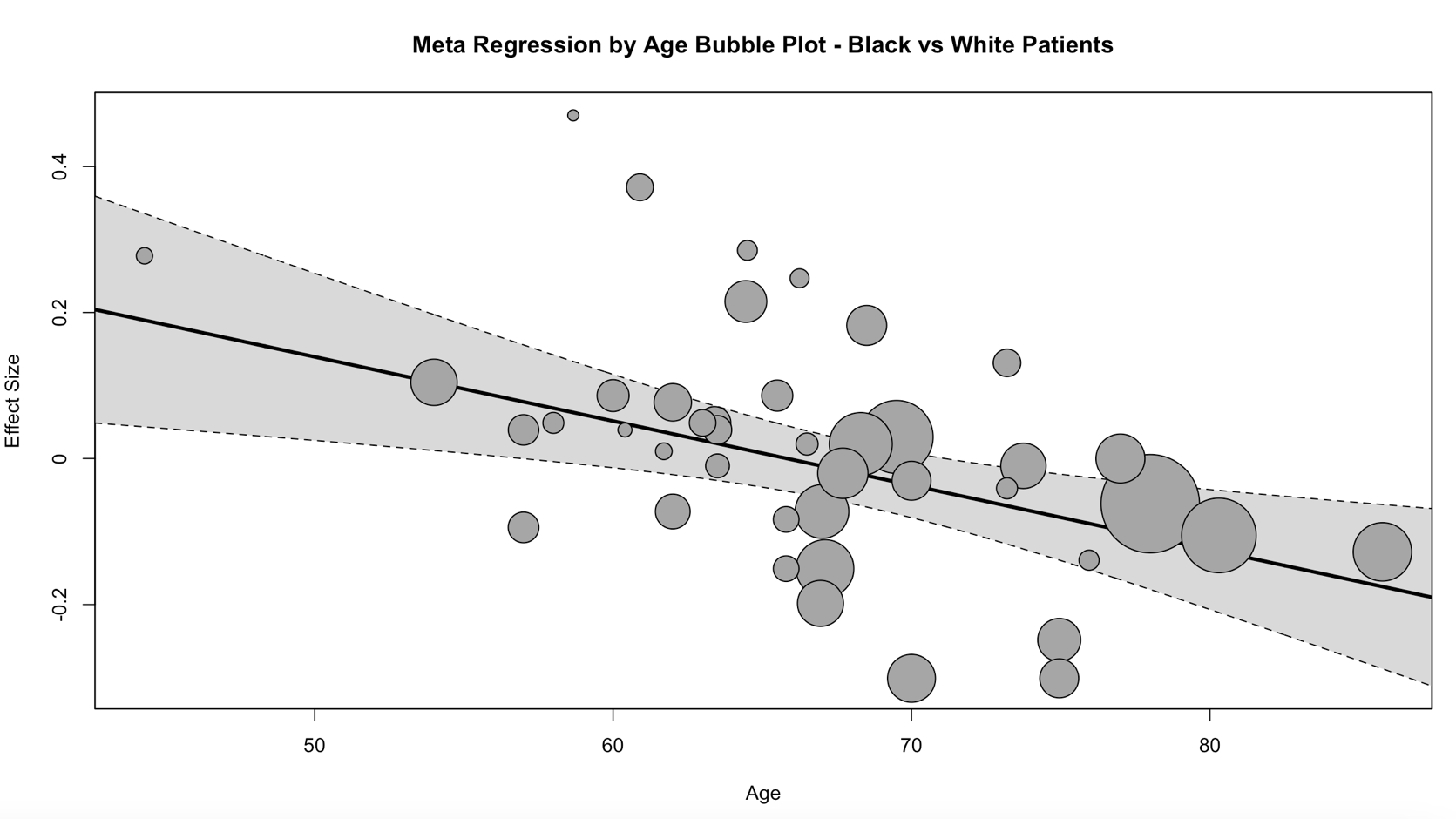
Supplementary Figure 8: Meta Regression bubble plot showing the relationship between mean age and effect size of Black versus White patient disparities in ACS mortality*

Abbreviations:

- ACS = Acute Coronary Syndrome
- SE = Standard Error

Footnotes:

- Each bubble represents an included study
- The solid line represents the regression line for the association between mean age and effect size
- The dashed lines represent the 95% confidence interval around the regression line
- Meta-regression showed statistically significant effect modification by age (p = 0.0033)
- As mean age increases, the effect size of ethnic disparities in ACS mortality between Black and White patients decreases, suggesting smaller differences between ethnic groups in older populations

*Supplementary Figure 9: Meta Regression bubble plot showing the relationship between mean age and effect size of Asian versus White patient disparities in ACS mortality*

*
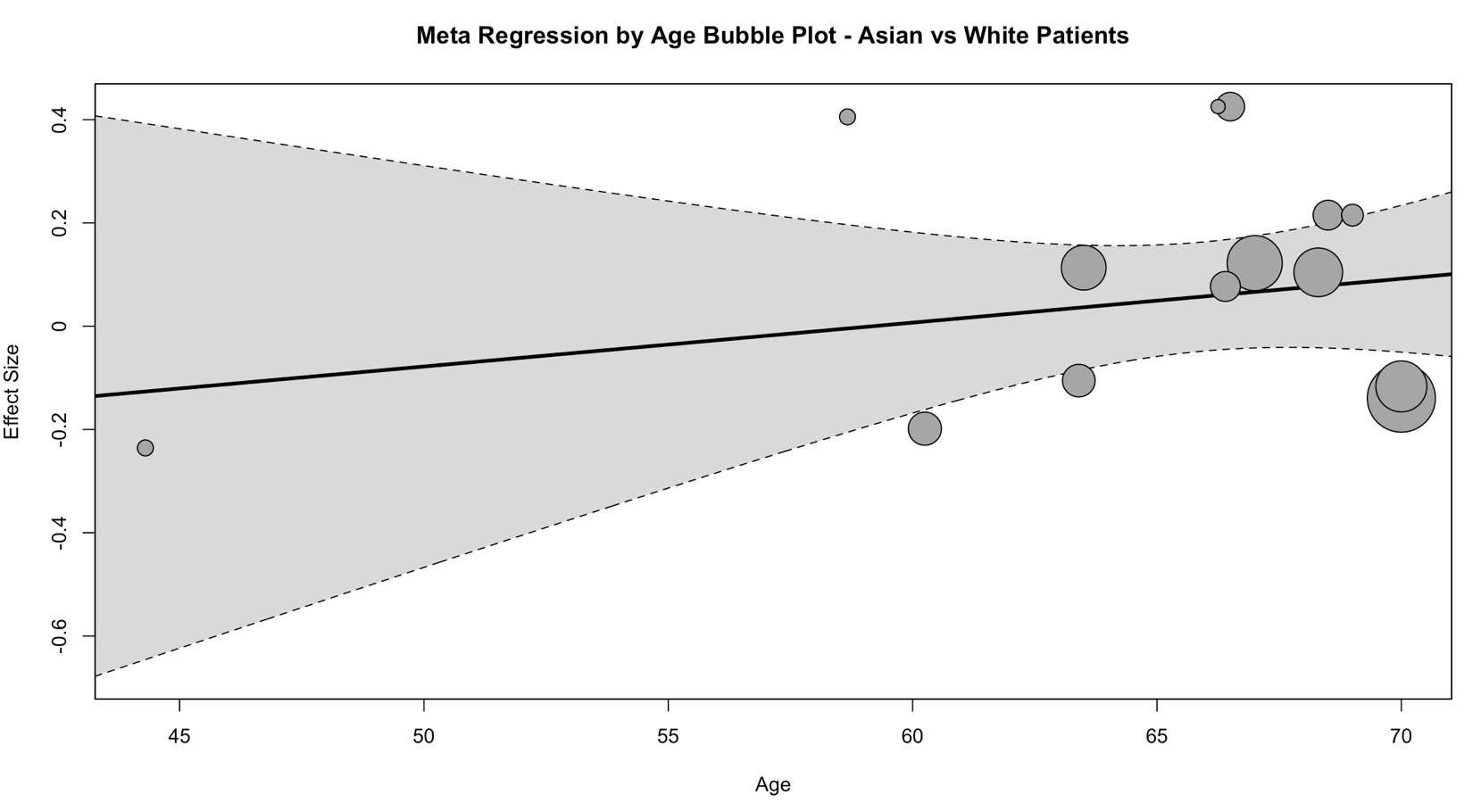
*

Abbreviations:

- ACS = Acute Coronary Syndrome
- SE = Standard Error

Footnotes:

- Each bubble represents an included study
- The solid line represents the regression line for the association between mean age and effect size
- The dashed lines represent the 95% confidence interval around the regression line
- Meta-regression showed no statistically significant effect modification by age (p = 0.48)

*Supplementary Figure 10: Meta regression bubble plot showing the relationship between year of publication and effect size of ethnic disparities in Black versus White patients ACS mortality*

*
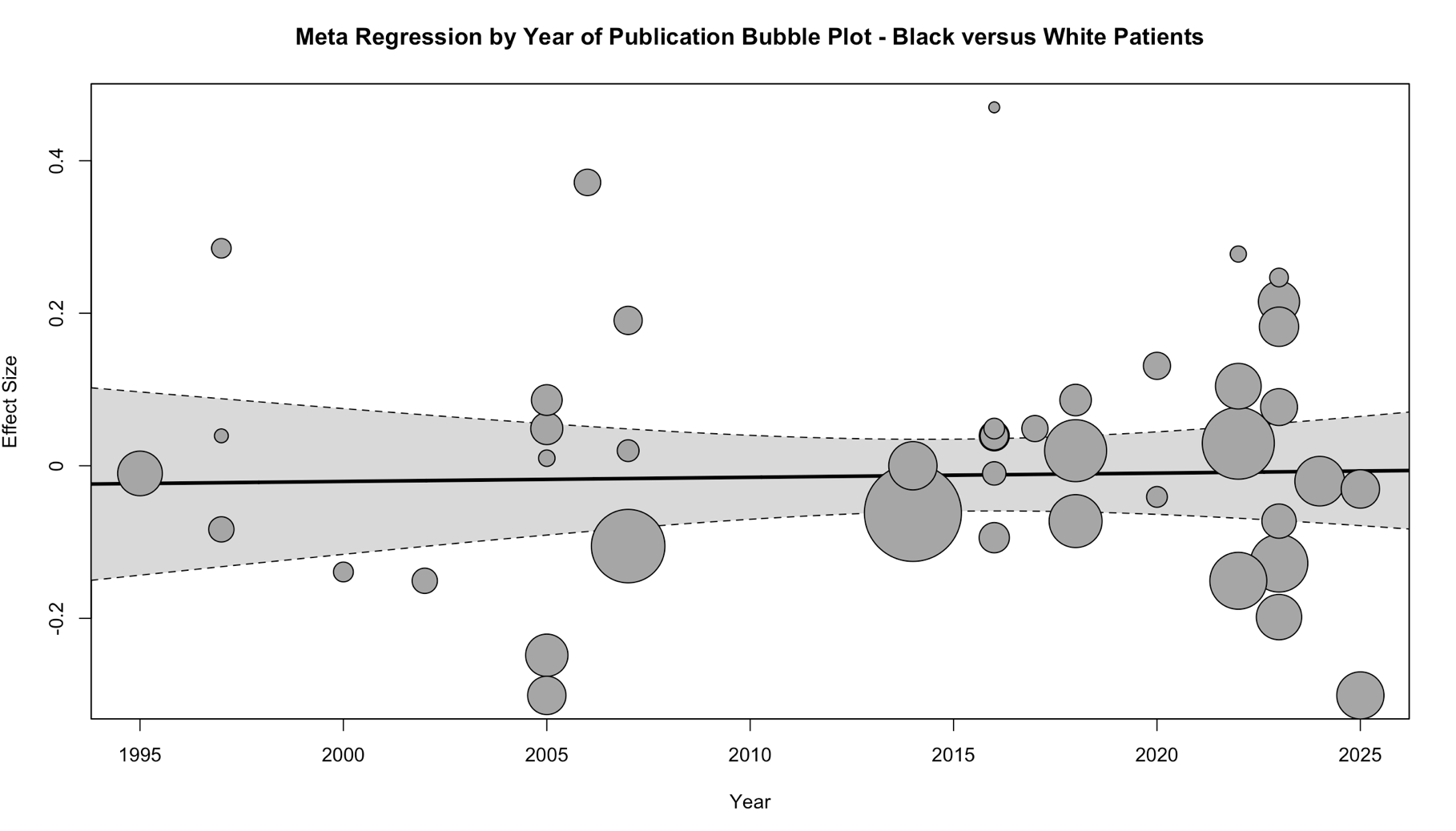
*

Abbreviations

- ACS = Acute Coronary Syndrome
- SE = Standard Error

Footnotes

- Each bubble represents an included study
- The solid line represents the regression line for the association between year of publication and effect size
- The dashed lines represent the 95% confidence interval around the regression line

Meta-regression by year of publication found no statistically significant association between publication year and effect size for ethnic disparities in Black versus White patients in ACS mortality (p = 0.85). This is seen in the bubble plot with the flat regression line and wide confidence intervals.

*Supplementary Figure 11: Meta regression bubble plot showing the relationship between year of publication and effect size of ethnic disparities in Asian versus White patients ACS mortality*


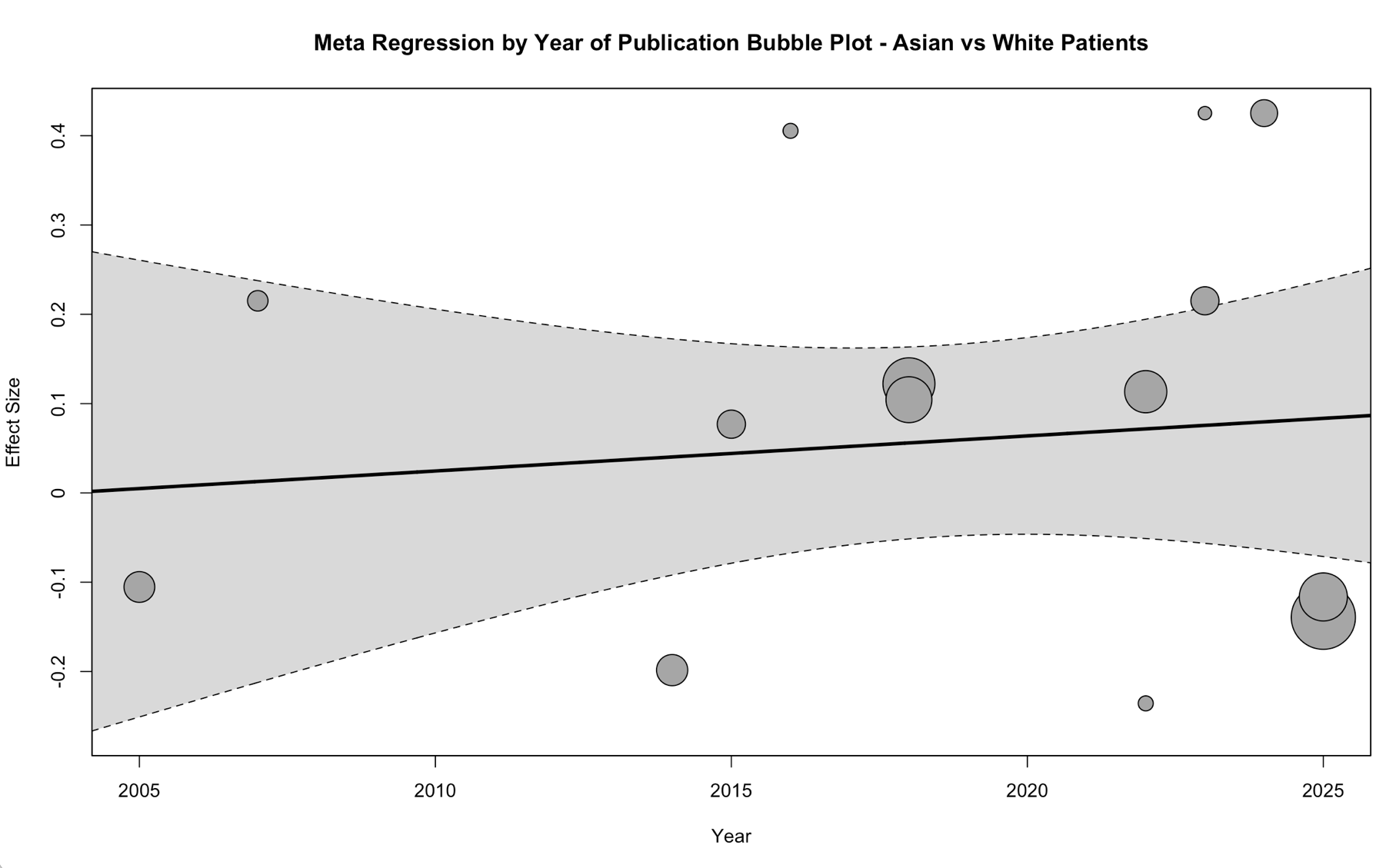


Abbreviations

- ACS = Acute Coronary Syndrome
- SE = Standard Error

Footnotes

- Each bubble represents an included study
- The solid line represents the regression line for the association between year of publication and effect size
- The dashed lines represent the 95% confidence interval around the regression line
- Meta-regression by year of publication found no statistically significant association between publication year and effect size for ethnic disparities in Asian versus White patients in ACS mortality (p = 0.65). This is seen in the bubble plot with the flat regression line and wide confidence intervals.
